# Development of Fluorescent Probes that Target Serotonin 5-HT_2B_ Receptors

**DOI:** 10.1038/s41598-017-11370-2

**Published:** 2017-09-07

**Authors:** Jhonny Azuaje, Paula López, Alba Iglesias, Rocío A. de la Fuente, José M. Pérez-Rubio, Diego García, Tomasz Maciej Stępniewski, Xerardo García-Mera, José M. Brea, Jana Selent, Dolores Pérez, Marián Castro, María I. Loza, Eddy Sotelo

**Affiliations:** 1 Centro Singular de Investigación en Química Biolóxica e Materiais Moleculares (CIQUS), Universidade de Santiago de Compostela, E-15782 Santiago de Compostela, Spain; 2 Departamento de Química Orgánica, Facultad de Farmacia, Universidade de Santiago de Compostela, E-15782 Santiago de Compostela, Spain; 3 Instituto de Farmacia Industrial (IFI), Universidade de Santiago de Compostela, E-15782 Santiago de Compostela, Spain; 4 Centro Singular de Investigación en Medicina Molecular e Enfermidades Crónicas (CIMUS), Universidade de Santiago de Compostela, E-15782 Santiago de Compostela, Spain; 5 PharmacoInformatics Group, Research Program on Biomedical Informatics (GRIB) PRBB, Barcelona, 08003 Spain; 6 Faculty of Chemistry, Biological and Chemical Research Centre, University of Warsaw, 02-093 Warsaw, Poland

## Abstract

Some 5-HT_2B_ fluorescent probes were obtained by tagging 1-(2,5-dimethoxy-4-iodophenyl)-propan-2-amine (DOI) with a subset of fluorescent amines. Some of the resulting fluorescent ligands showed excellent affinity and selectivity profiles at the 5-HT_2B_ receptors (e.g. **12b**), while retain the agonistic functional behaviour of the model ligand (DOI). The study highlighted the most salient features of the structure-activity relationship in this series and these were substantiated by a molecular modelling study based on a receptor-driven docking model constructed on the basis of the crystal structure of the human 5-HT_2B_ receptor. One of the fluorescent ligands developed in this work, compound **12i**, specifically labelled CHO-K1 cells expressing 5-HT_2B_ receptors and not parental CHO-K1 cells in a concentration-dependent manner. **12i** enables imaging and quantification of specific 5-HT_2B_ receptor labelling in live cells by automated fluorescence microscopy as well as quantification by measurements of fluorescence intensity using a fluorescence plate reader.

## Introduction

The biogenic amine serotonin, 5-hydroxytryptamine (5-HT), is one of the most versatile chemical messengers in the central and peripheral nervous systems^1^. In addition to its well-known role as a neurotransmitter^2^, regulating virtually all brain functions and neurophysiological processes, 5-HT controls critical functions^3^ within cardiovascular, pulmonary, gastrointestinal and genitourinary systems. Consequently, serotonin has been implicated in the etiology of numerous disease states^4^ (e.g., depression, anxiety, schizophrenia, obsessive-compulsive and panic disorders, migraine, hypertension, pulmonary hypertension, eating disorders, vomiting and irritable bowel syndrome). The large diversity of functions of serotonin is paralleled by the pharmacological complexity of serotonin receptors^5^. Of the 14 mammalian 5-HT receptor subtypes, all but one (5-HT_3_) belong to the super-family of G protein-coupled-receptors (GPCRs)^5, 6^, which mediate most of the serotonin-based signalling network by receiving and modulating complex information^6, 7^.

The 5-HT_2_ receptor family comprises three closely related receptor subtypes^8, 9^, namely 5-HT_2A_, 5-HT_2B_, and 5-HT_2C_, that are the molecular targets of prominent drugs acting in different therapeutic areas^1–4, 9–11^ (e.g., schizophrenia, depression, hypertension, anxiety). 5-HT_2_ receptor subtypes mediate many of the central and peripheral physiological functions of serotonin^1–4^ and they couple preferentially to G_q/11_ to increase the hydrolysis of inositol phosphates (IPs) and elevate cytosolic Ca^2+^. This similar functional behaviour is supported by a high structural homology^8, 9^. 5-HT_2A_, 5-HT_2B_, and 5-HT_2C_ receptors share approximately 46–50% amino acid sequence identity, with the homology being higher than 70% within the transmembrane domains (which contain the 5-HT_2_ binding pocket)^8, 9^. Accordingly, the development of highly selective ligands that target a particular 5-HT_2_ receptor subtype constitutes a considerable challenge. The recent resolution of the crystal structure of the 5-HT_2B_ receptor^7^ provides new opportunities for the rational discovery of novel small-molecule modulators of 5-HT receptors.

The 5-HT_2B_ subtype remains one of the most attractive and enigmatic receptors amongst the 5-HT receptor superfamily^12, 13^, with key functional, signalling, and regulatory aspects remaining ambiguous. The involvement of this receptor in the development of migraine^14^, the modulation of the 5-HT transport system^15^, and the rewarding and reinforcing effects of the widely abused drug ecstasy (3,4-methylenedioxy-N-methylamphetamine, MDMA) has been validated^16^. 5-HT_2B_ receptor also participate in other relevant processes, particularly in the cardiovascular system where it regulates cardiac development and cardiomyocyte proliferation and survival^17, 18^. 5-HT_2B_ activation has been associated with diverse pathologies^19, 20^ (e.g., cardiac hypertrophy and pulmonary hypertension). It has been shown that 5-HT_2B_ activation, along with the inhibition of serotonin transporters, plays a significant role in the pathogenesis of serotonin-induced valvular abnormalities^20–22^. In line with these observations, it has been demonstrated that norfenfluramine (a metabolite of the antiobesity drug fenfluramine that exhibits potent 5-HT_2B_ agonist activity) and other 5-HT_2B_ agonists used in the treatment of migraine induce valvular heart disease^23–25^. Similarly, it was recently verified that the use of the antiparkinsonian dopaminergic agonists cabergolide and pergolide (both of which also exhibit high 5-HT_2B_ agonism) is associated with mitral, aortic, and tricuspid valvular heart disease^26, 27^. Accordingly, drugs that are able to activate 5-HT_2B_ receptors and/or increase circulating serotonin levels are considered to be potential valvulophatic inducers and, as a consequence, 5-HT_2B_ agonism is considered a dangerous off-target effect^22, 27–29^, thus hampering the promising therapeutic potential of 5-HT_2B_ receptor^28^. However, our understanding of the role of 5-HT_2B_ receptor and an in-depth knowledge of the processes triggered by ligand−5-HT_2B_ receptor interactions in living cells are still limited due to the lack of *ad hoc* molecular probes.

The introduction of fluorescence-based techniques has progressed the study of GPCR pharmacology to the single cell level^30^. Fluorescent molecular probes have proven to be valuable tools that offer a wealth of relevant evidence^31, 32^, particularly the mapping or identification of ligand binding sites, ligand binding mechanisms, the physical nature of the binding pocket, the movement and internalization of receptors in living cells, and the localization and visualization of labelled receptors. Furthermore, fluorescent probes represent a safer, less expensive and faster alternative to radioligands. The extensive application of these techniques to GPCR research demands the development of fluorescently labelled GPCR ligands that have appropriate photochemical and pharmacological properties. A number of fluorescent probes have been described for 5-HT_1A_^33^, 5-HT_3_^34^ and 5-HT_6_^35^ receptors but, to the best of our knowledge, reports concerning fluorescently-tagged 5-HT_2_ ligands have not been published to date. As part of a project aimed at developing molecular probes for the study of the 5-HT_2_ receptor family, we report here the development and optimization of fluorescent tools that selectively target 5-HT_2B_ receptors. The new ligands combine good fluorescence properties with satisfactory affinity and selectivity; accordingly, these molecular probes can contribute to a better understanding of the physiological and pathological implications of 5-HT_2B_ receptors.

## Results and Discussion

1-(2,5-Dimethoxy-4-iodophenyl)-propan-2-amine (DOI)^36^ was selected as the reference ligand to develop the fluorescent probes designed within this study. DOI is a partial agonist derived from the amphetamine chemotype and it is one of the most useful and better pharmacologically characterized molecular tools for the study of the 5-HT_2_-receptor family^37, 38^. In addition, the [^125^-I]-R-DOI radioligand is recognized as a reference standard for high throughput screening campaigns at 5-HT_2_ receptors^39, 40^. Recent findings have shown that DOI produces a super-potent (≈15 pM) blockade of the pro-inflammatory effects of tumor necrosis factor alpha (TNF-α) in primary aortic smooth muscle cells and animal models^41, 42^. These findings, which provided new evidence about the role of 5-HT_2A_ receptors in inflammation, increase the demand for DOI-based fluorescent probes. For this study, it was decided to employ racemic DOI as reference, accordingly all molecular probes were synthesized and tested as racemates.

**Figure 1:**
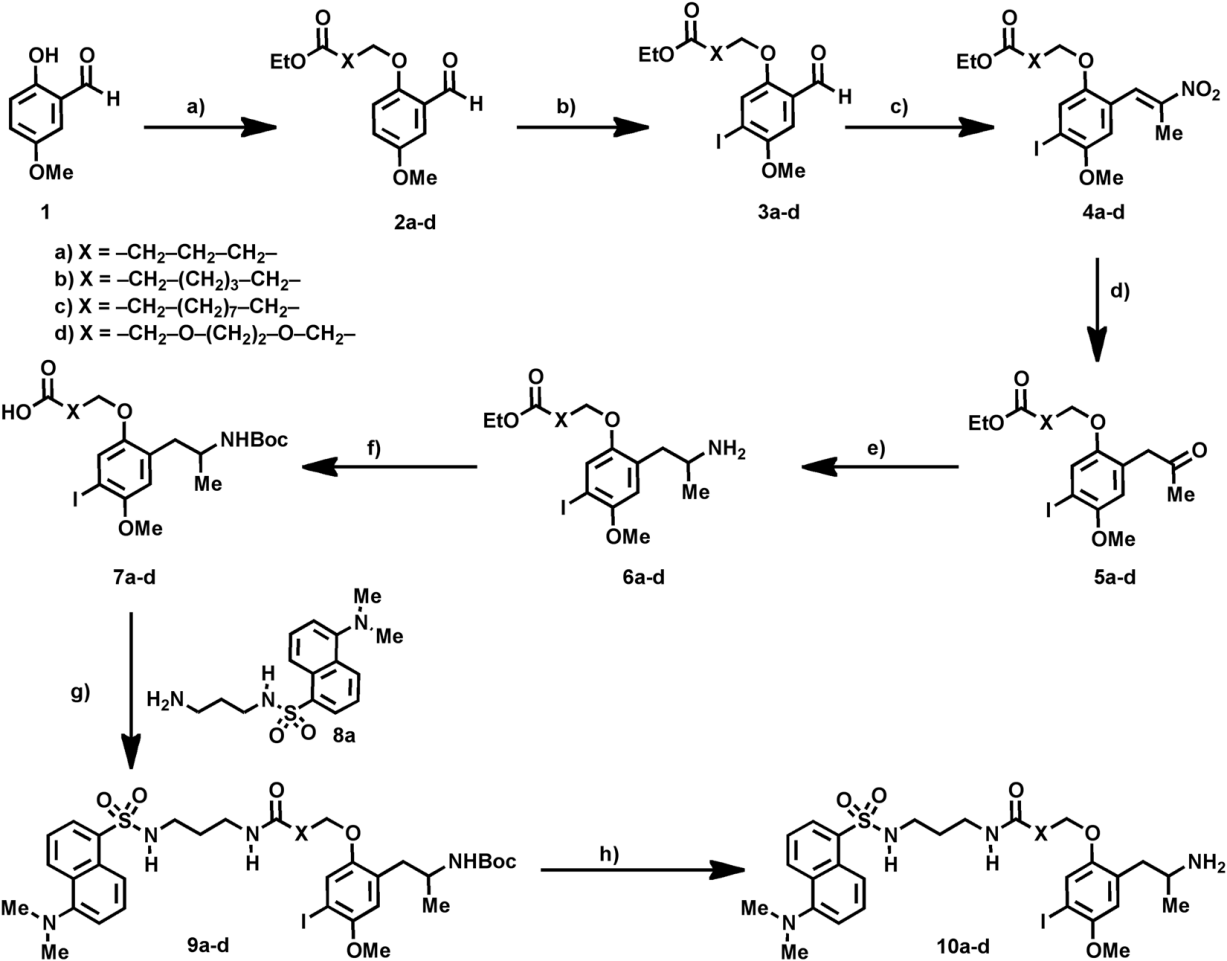
Synthesis of the target fluorescent DOI analogues^44–48^. Reagents and conditions: (**a**) K_2_CO_3_, R–X, MeCN, 80 °C, 8 h. (**b**) I_2_, THF, rt, 24 h. (**c**) Me-NO_2_, AcOH, 100 °C, 6 h. (**d**) Fe, AcOH, 100 °C, 12 h. (**e**) NH_4_OAc, NaCNBH_4_, THF, 2 h. (**f**) (Boc)_2_O, THF, 0 °C, 3 h. (**g**) DCC, DCM, rt, 12 h (70–93%). (**h**) HCl/Dioxane, DCM, 0 °C, 2 h (50–78%).

DOI is a well-recognized specific 5-HT_2_ ligand that, notwithstanding, exhibits weak selectivity within the 5-HT_2_ receptor family (5-HT_2A_, 5-HT_2B_, 5-HT_2C_)^36–38^. Previous reports have evidenced the introduction of functionalized alkyl chains on the methoxy group at position 2 of the phenyl ring in DOI produces an increased 5-HT_2B_ selectivity^43^. This structure-activity relationship trend encouraged us to explore the use of DOI-based derivatives for the development of fluorescent ligands for the 5-HT_2B_ receptor. A set of acid-functionalized DOI derivatives (**7a**–**d**) bearing variable spacers on the oxygen atom at position 2 was synthesized (Fig. 1) according to previously described procedures^44–48^. With the aim of carrying out a preliminary evaluation of the biological repercussions of the different spacers, precursors **7a**–**d** were transformed into the corresponding fluorescent derivatives **10a**–**d** by reacting with a model dansyl amine (**8a**) and subsequent cleavage of the Boc groups (Fig. 1).

**Figure 2:**
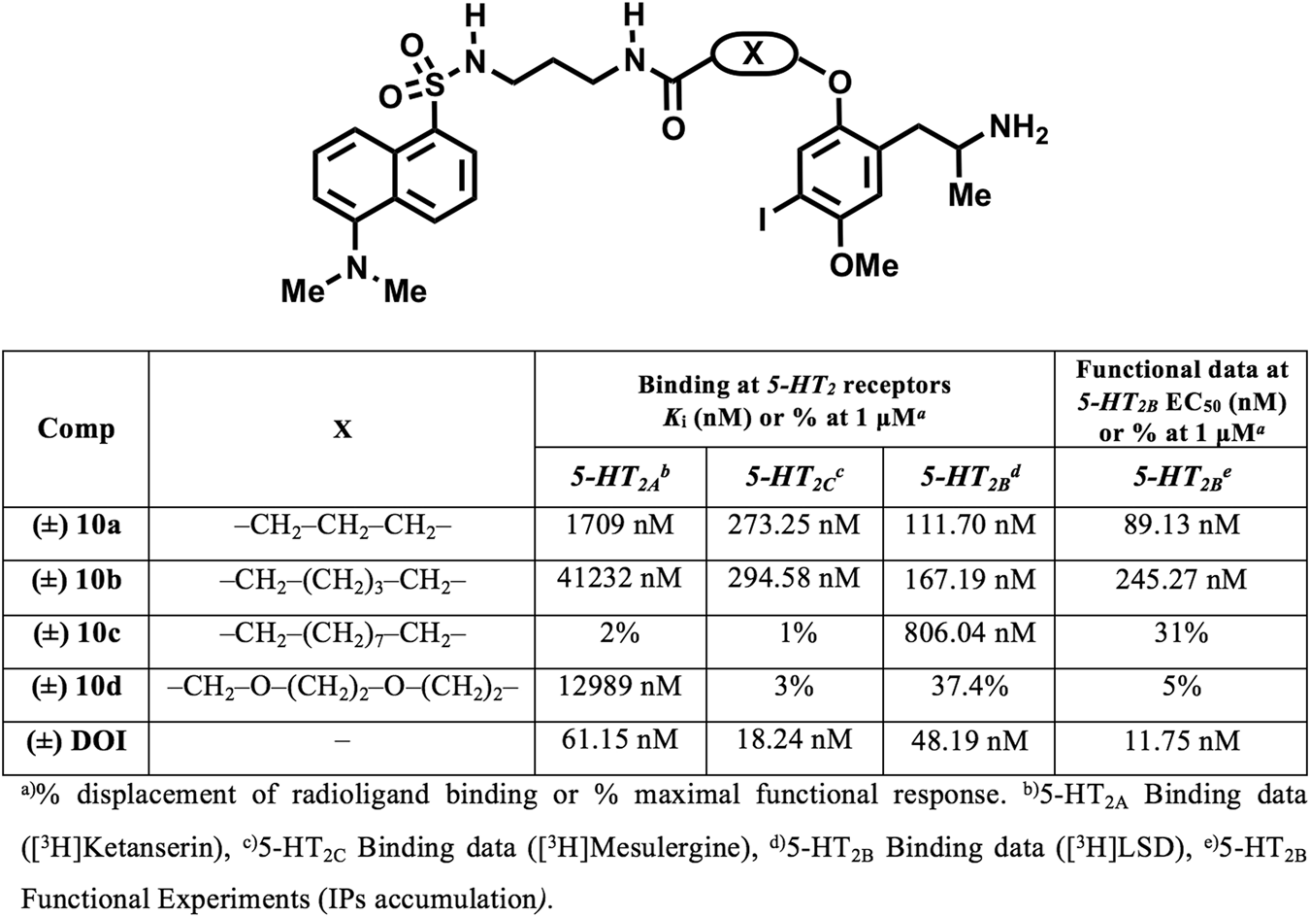
Structural and pharmacological data at 5-HT_2_ receptors of model dansyl probes bearing different linkers (**10a**–**d**).

The fluorescent probes **10a**–**d** contain the 1-(2,5-dimethoxy-4-iodophenyl)-propan-2-amine scaffold and the dansyl chromophore linked through variable spacers that differ in both length and composition (Fig. 2). The results of the pharmacological evaluation of the model fluorescently-tagged amides **10a**–**d** at the 5-HT_2_ receptor family (5-HT_2A_, 5-HT_2B_, 5-HT_2C_)^49, 50^ are presented in Fig. 2. All compounds were evaluated in binding experiments (see experimental part) and the results were compared with the biological activity determined for the reference ligand (DOI). Additionally, the functional activities (IPs accumulation)^51^ of the compounds at the 5-HT_2B_ receptor were also determined (Fig. 2).

In agreement with previous findings^43^, it was verified that the introduction of alkyl chains on the oxygen atom at position 2 of the phenyl ring of the amphetamine scaffold produces derivatives that exhibit incipient 5-HT_2B_ subtype selectivity (**10a**–**d**, Fig. 2). The drop observed in the 5-HT_2A_ affinity is particularly noteworthy as this is in the micromolar range for all of the dansyl probes tested. Another remarkable trend identified on considering the biological data (Fig. 2) obtained for DOI derivatives bearing dansyl as a fluorescent tag (**10a**–**d**) is the importance of the spacer group linking the pharmacophore and the fluorophore. It was observed that elongation of the spacer has a negative effect on both the affinity and activity at the 5-HT_2B_ receptor (Fig. 2), with the fluorescent probe bearing the propyl group (**10a**) displaying the most attractive affinity and selectivity profiles.

**Figure 3:**
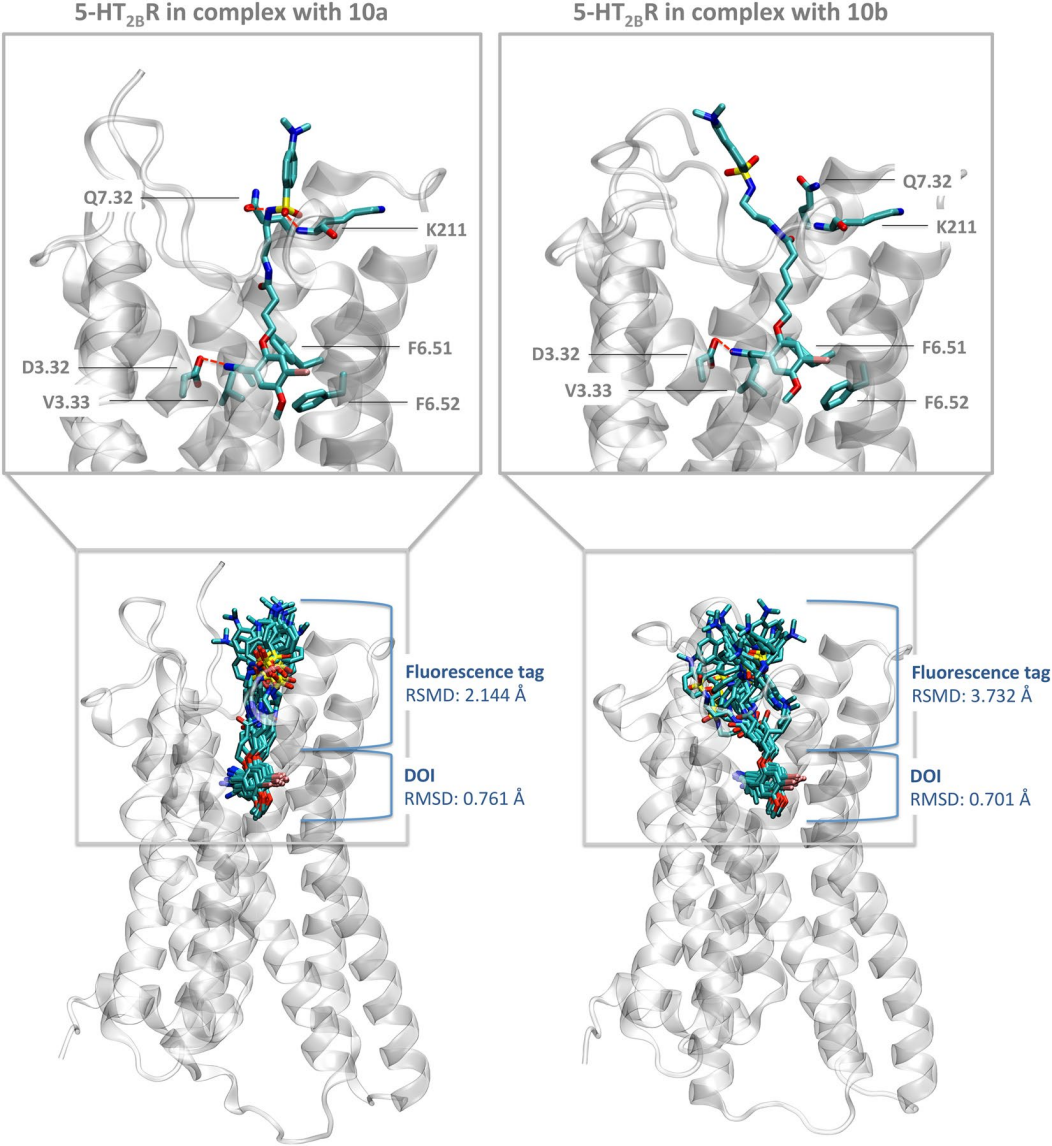
Three-dimensional complexes of the fluorescent probes **10a** (left) and **10b** (right) bound to the 5-HT_2B_ receptor obtained from extended molecular dynamics simulation with a total time of 1.6 μs (2 times × 800 ns). The upper panel highlights ligand-receptor interactions of a representative structure. The lower panel includes information about the dynamic properties and stability of the ligand binding by depicting frames each 50 ns along a total simulation time of 800 ns. The RMSD values are calculated for DOI or the fluorescent tag with respect to the average structure at 800 ns.

In an effort to shed light on the molecular determinants for the binding of the novel fluorescent probes, we used the recent crystallized structure of the 5-HT_2B_ receptor (PDB ID: 4IB4)^7^ to create three-dimensional models of this receptor in complex with two fluorescent derivatives with different linker lengths: compound **10a** (3 carbon linker) and **10b** (5 carbon linker). The resulting complexes were embedded in a physiological environment consisting of a hydrated lipid bilayer and then subjected to extended molecular dynamics simulations with a total simulation time of 1.6 µs. A common binding pose was obtained for the DOI fragment for both simulated systems and this fragment is inserted deep into to the 5-HT_2B_ receptor (Fig. 3, inset top panel). The protonated nitrogen of DOI establishes a strong electrostatic interaction with D3.32 whereas the aromatic ring is sandwiched in a hydrophobic environment formed by a valine in position 3.33 and two phenylalanines in positions 6.51 and 6.52. This tight binding results in a stable pose along the simulation, as reflected by low RMSD values of 0.761 Å (compound **10a**) and 0.703 Å (compound **10b**) with respect to the average structure (Fig. 3, lower panel). In contrast, the fluorescent tag that is linked to position 2 of the aromatic ring and reaches towards the extracellular side of the 5-HT_2B_ receptor shows much higher dynamic variability around the average structure, with an RMSD of 2.144 Å for ligand **10a** and 3.732 Å for compound **10b**. The general higher fluctuation for both fluorescent probes is due to interaction with areas of higher flexibility in the extracellular loop region of the receptor. This situation is inevitable as the fluorescent tag needs to reach sufficiently far out of the receptor in order to avoid undesired quenching interactions with the receptor, which would give rise to a non-functional fluorescence probe.

Despite this higher fluctuation, it was found that during the course of the simulation compound **10a** (3 carbon linker) formed frequent polar interactions with the backbone of ECL 2 and Q7.32 *via* the SO_2_ group (Fig. 3, top left panel), which result in a stabilization of the fluorescent tag (RMSD 2.144 Å, bottom left panel). Such polar stabilization is reduced for compound **10b** because the longer linker architecture (5 carbon linker) is not optimal for allowing this polar interaction. Such reduced stabilization could be a plausible explanation for the reduced binding affinity observed for compound **10b** (5 carbon linker, K_*i*_: 167.19 nM) compared to compound **10a** (3 carbon linker, K_*i*_: 111.70 nM). The model developed here predicts that compounds with a longer linker have reduced stabilizing interactions in the extracellular loop regions and therefore exhibit lower binding affinity. This prediction is consistent with the other experimental data. For example, compound **10c** (9 carbon linker, K_*i*_ 806.04 nM) has a markedly lower binding affinity compared to compound **10a** (3 carbon linker, K_*i*_: 111.70 nM) and **10b** (5 carbon linker, K_*i*_ 167.19 nM).

**Figure 4:**
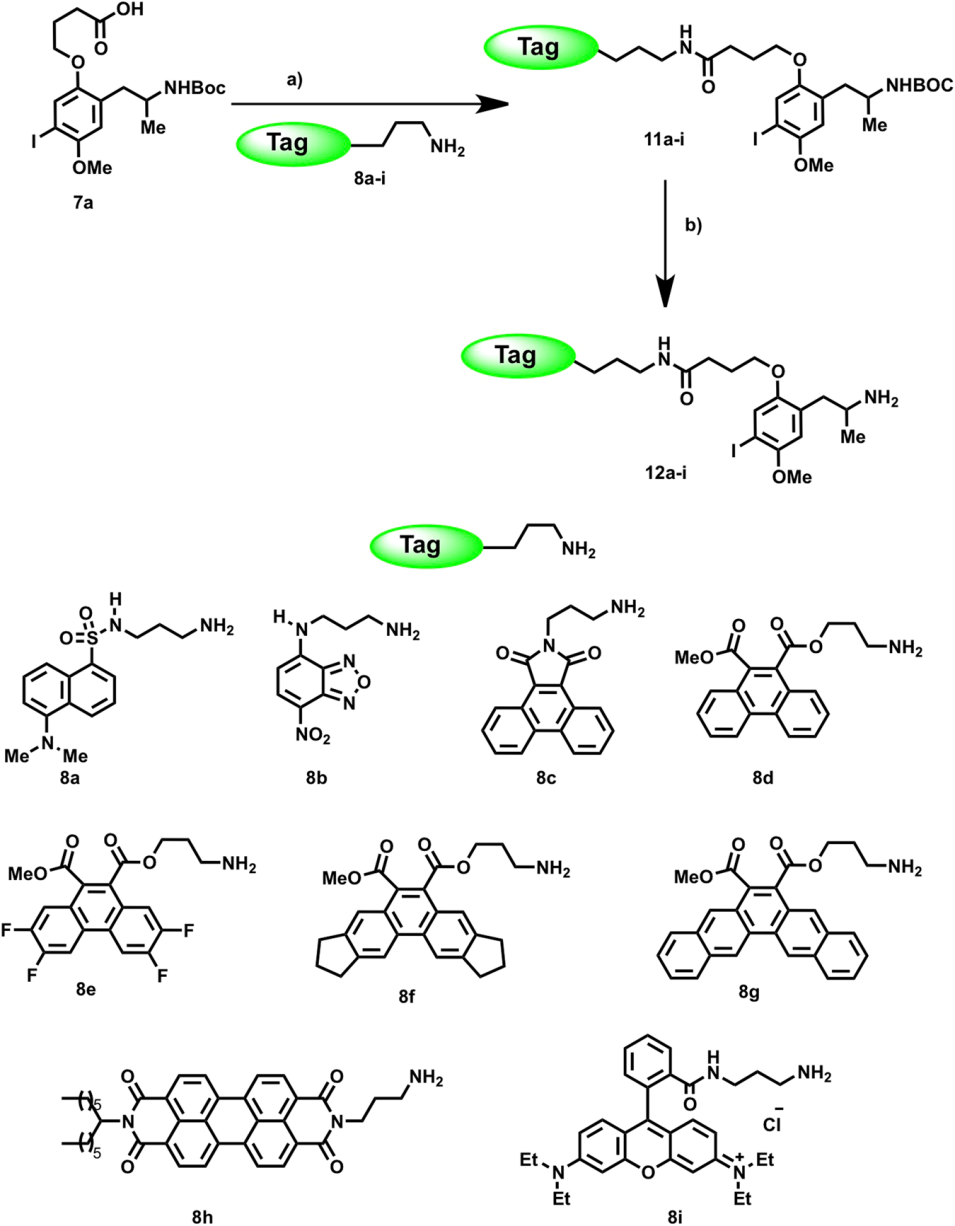
Synthesis of the target fluorescent DOI analogues **12a**–**i** and structures of the fluorescent amine precursors (**8a**–**i**). Reagents and conditions: (**a**) DCC, DCM, rt, 12 h (70–93%). (**b**) HCl/Dioxane, DCM, 0 °C, 2 h (50–78%).

The propyl spacer group present in the most pharmacologically appealing fluorescent probe identified (**10a**) was fixed for subsequent fluorophore exploration. Accordingly, the carboxylic acid **7a** was combined with a set of 9 propylamine-functionalized fluorescent tags (Fig. 4) by employing classical coupling experimental conditions (Fig. 4)^44–48^. The selection of fluorescent amines (**8**) embraced not only classical fluorescent tags (e.g., **8a**,**b** or **8i**) but also several new fluorescent scaffolds (e.g., **8c**–**h**). The employed tags (**8**) incorporate fluorescent groups with relatively small to large molecular volumes, thus providing a collection of probes with diverse structural and photophysical properties. Some of the non-conventional fluorophores were chosen on the basis of their complementary absorption/emission fluorescence spectra, since they could be employed as donor and acceptor pairs for the implementation of fluorescence resonance energy transfer (FRET) studies in the near future. Amines **8d**–**8g** were prepared from the corresponding dimethyl phenanthrene-9,10-dicarboxylates (or pentaphene-6,7-dicarboxylate, for **8g**), these aromatic *o-*diesters were synthesized by palladium-catalyzed [2 + 2 + 2] cycloaddition of arynes^52, 53^ with dimethyl acetylenedicarboxylate (see supporting information). The experimental details for the syntheses of compounds **12** are outlined in Fig. 4. Briefly, the carboxylic acid **7a** was reacted with different fluorescent amines (**8a**–**i**) employing DCC as a coupling reagent to give the amides **11**. Finally, the protecting group (BOC) was removed by treatment with TFA to afford the fluorescent DOI-based probes **12a**–**i**. Details on the synthesis, structural and photo-physical characterization of the obtained fluorescent probes are described in the experimental part and in Fig. 4.

**Figure 5:**
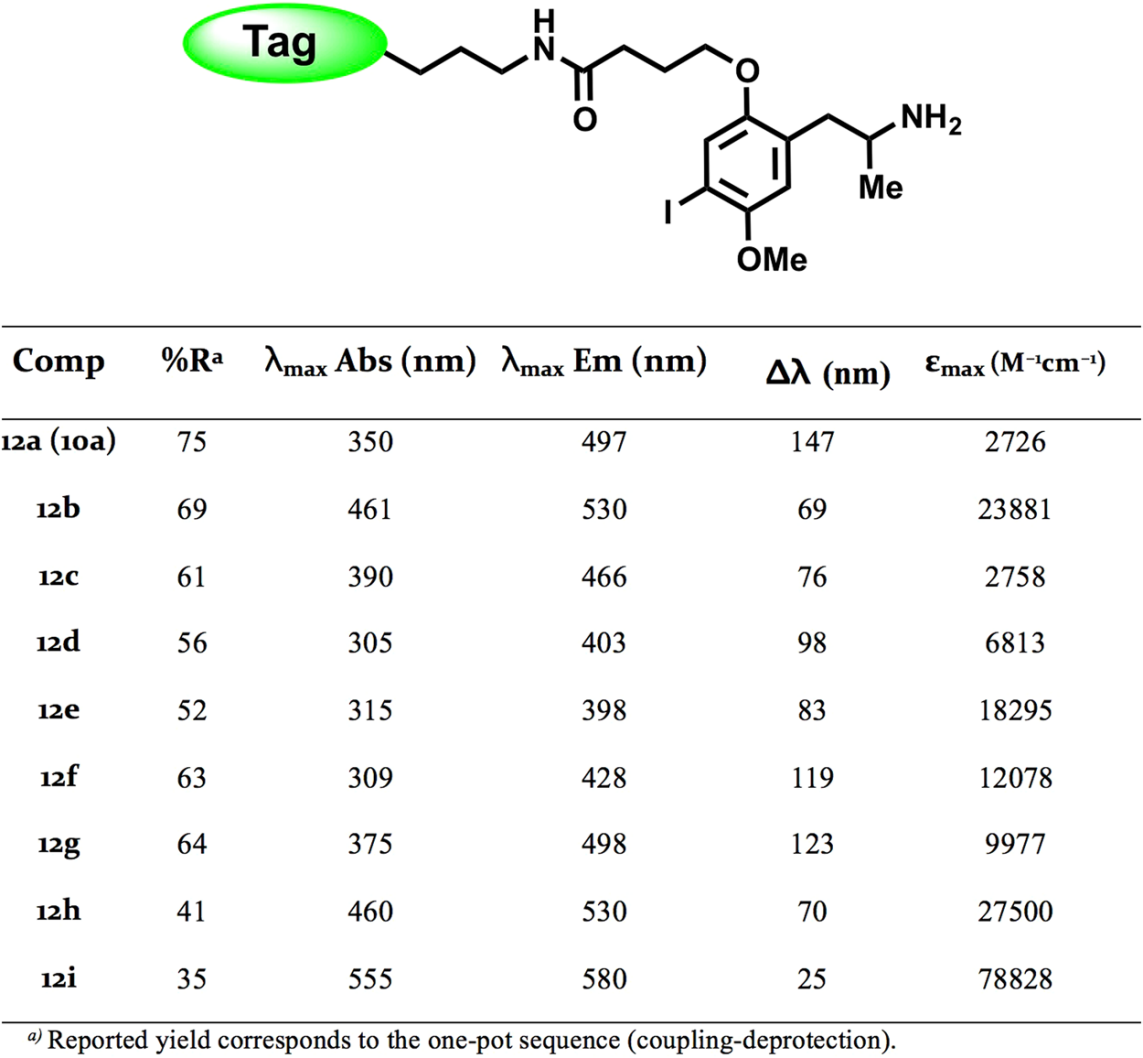
Photophysical properties of fluorescent DOI probes **12a**–**i**.

The absorption and emission spectra of the fluorescent 5-HT_2_ ligands (**10** and **12**) were determined on 10 μM solutions. The most relevant fluorescent properties are collected in Fig. 5. All of the synthesized derivatives emitted fluorescence in the visible region of the spectrum, with selected fluorescent tags (Fig. 4) providing distinctive properties and excitation wavelengths. The excitation wavelengths varied from 305 to 555 nm, whereas those for emission varied from 398 to 580 nm. Most of the compounds showed satisfactory Stokes shift values (Fig. 5).

**Figure 6:**
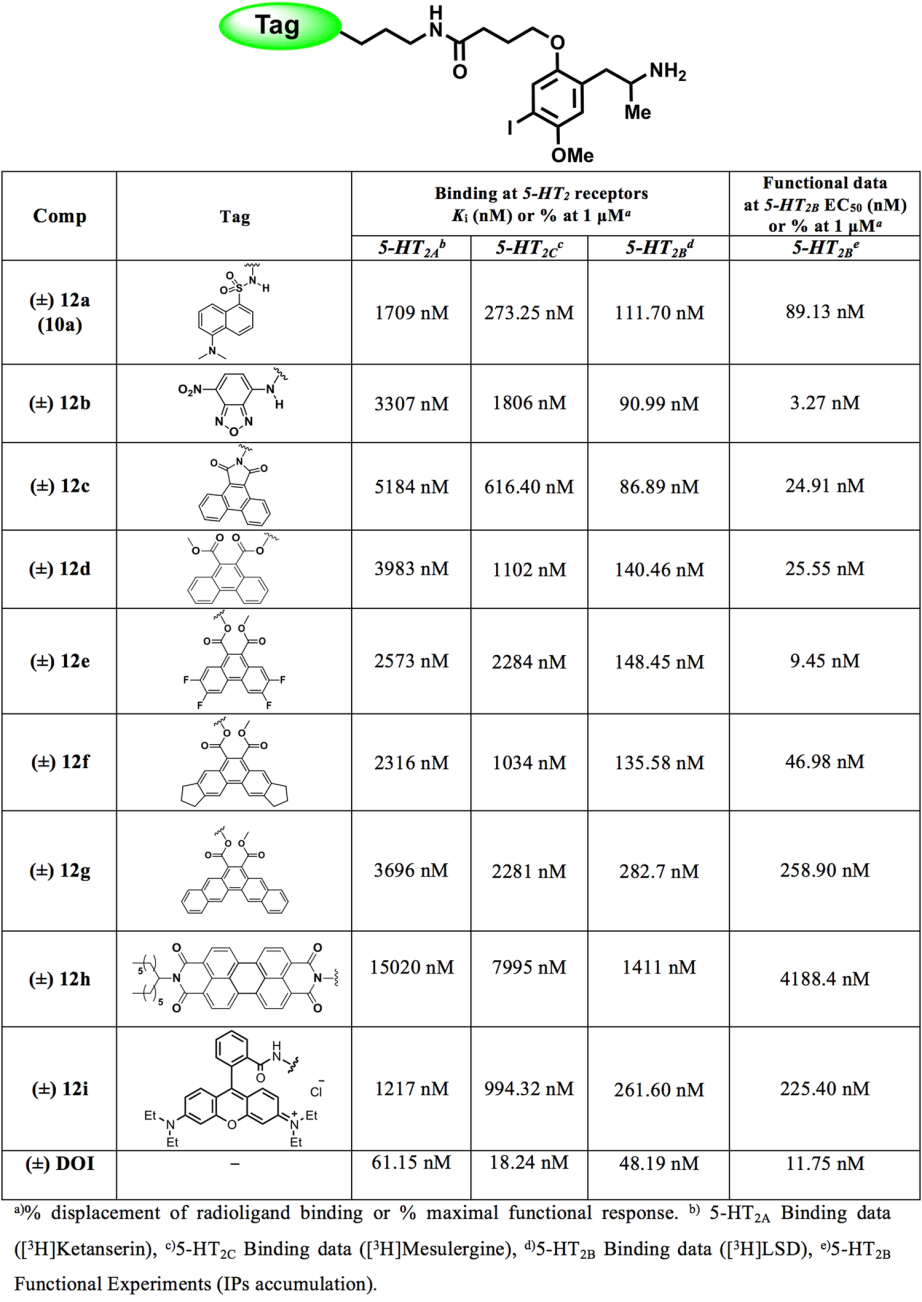
Structures and pharmacological data at 5-HT_2_ receptors for the DOI-based fluorescent probes **12a–i**.

**Figure 7:**
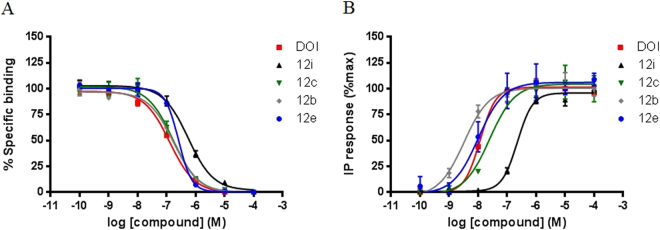
Concentration-response curves obtained for representative ligands and DOI in binding experiments (**A**) and in functional studies (**B**).

As for the first series described above, the pharmacological characterization of the resulting fluorescent ligands (**12**) was performed at two levels. Firstly, the *in vitro* affinity was assessed by radioligand binding assays at the three 5-HT_2_ receptor subtypes. Subsequently, the functional activity at the 5-HT_2B_ receptors was evaluated (IPs accumulation). The data are collected in Fig. 6 and some of the representative curves obtained are shown in Fig. 7. It can be observed that all compounds stimulated IPs accumulation in a concentration-dependent manner, with EC_50_ values in the nanomolar range. These data confirm that the novel ligands retain the agonist behaviour exhibited by the model ligand (DOI).

**Figure 8:**
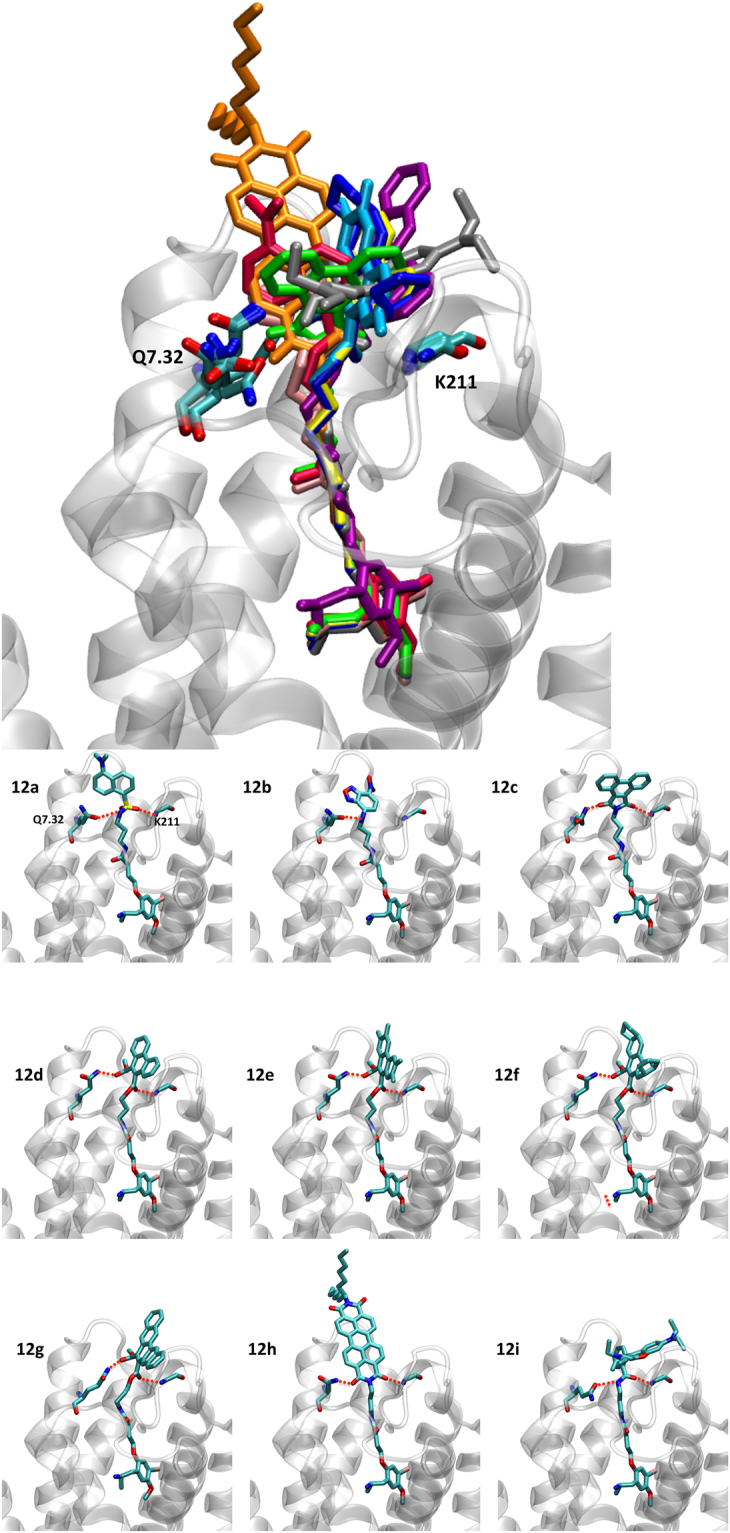
(**A**) Superimposition of docking poses of compounds **12a**–**i** for the 5-HT_2B_ receptor (PDB ID: 4IB4) with following color code: **12a** - red, **12b** - pink, **12c** - green, **12d** - yellow, **12e** - cyan, **12f** - dark blue, **12g** - purple, **12h** - orange and **12i** - silver. (**B**) Individual docking poses for the probes **12a**–**i** bound to the 5-HT_2B_ receptor (PDB ID: 4IB4).

Examination of the pharmacological data contained in Fig. 6 reveals the identification of some highly attractive fluorescent ligands (e.g., **12b**–**f**). Most of the fluorescent probes retain the incipient 5-HT_2B_ selectivity profile observed for the former member of the series (**10a**). Taking advantage of the existing X-ray structure, we performed docking followed of molecular probes from the **12** series (**12a** to **12i**) into the 5-HT_2B_ receptor (PDB ID: 4IB4). All probes have overlapping docking poses (Figs 3 and 8) forming common interactions of the DOI fragment with residues D3.32, V3.33, F6.51 and F6.52 in the orthosteric binding pocket, as previously observed in our molecular dynamics simulations. In addition, we find that two residues, Q7.32 and the backbone of K211 at the extracellular loop clamp the polar part of the fluorophore tag (highlighted as red dashed lines in Fig. 8). The linker length determines the position of the fluorophore and its polar region with respect to the polar extracellular loop region. As discussed above, it is likely that longer linkers limit such favourable polar interaction. The obtained binding modes (Fig. 8) provide a potential explanation for affinity differences of studied compounds. According to our models, it appears that solvent exposure is an important factor for binding affinity towards the 5-HT_2B_ receptor. Comparing dyes **12a** to **12h** (Fig. 8) suggests that increased and unfavourable exposure of complex and hydrophobic probes that reach out into the solvent seems to be responsible for a drop of 5-HT_2B_ affinity.

The obtained data support the benefits of introducing the acid-functionalized linker on the oxygen atom at position 2 of the phenyl ring of the model ligand (DOI), but also highlight the need for the optimized alkyl spacer (X = CH_2_−CH_2_−CH_2_). It should be noted that most of the fluorescent probes exhibited weak affinity (typically micromolar range) at the 5-HT_2A_ and 5-HT_2C_ receptors, while the functional experiments confirmed their agonistic behaviour (Fig. 7). Moreover, a progressive drop in 5-HT_2B_ affinity was observed when the molecular complexity of the dye increased (e.g. cpds **12g**–**i**). Of the probes obtained, the 7-nitrobenz-2-oxa-1,3-diazole (NBD) labelled DOI ligand (**12b**) warrants particular attention as it is slightly less potent (*K*_i_ = 90.99 nM) than the reference ligand (DOI, *K*_i_ = 48.19 nM) while exhibit an excellent selectivity (≥20-fold) for the 5-HT_2B_ receptor subtype. Although the NBD ligand (**12b**) has the most attractive pharmacological profile, the rhodamine derivative **12i** showed the most promising properties of the series from the photophysical point of view.

Having finished the preliminary photophysical and pharmacological characterization of the fluorescent derivatives **12a**–**i**, their potential to specifically label 5-HT_2B_ receptors in live cells was evaluated. Chinese hamster ovary K1 (CHO-K1) cells and CHO-K1 cells stably expressing the human serotonin 5-HT_2B_ receptor (CHO-K1-5-HT_2B_), growing in 96-well plates, were incubated with at least three different concentrations of each compound for varying times and, after compound removal and washing of the cells, plates were subjected to fluorescence microscopy using a high content imaging instrument and the appropriate excitation and emission filter set for each compound.

Unfortunately, substantial specific labelling of 5-HT_2B_ receptors was not observed for compounds **12a**–**h** under any of the conditions evaluated. For these compounds, the cellular fluorescence intensities were similar in CHO-K1-5-HT_2B_ and in parental untransfected control CHO-K1 cells (data not shown). This non-specific labelling of cells independently of 5-HT_2B_ expression might arise due to non-specific membrane binding or membrane penetration of the fluorescent ligands, as observed previously for other fluorescent probes for GPCRs^54^.

**Figure 9:**
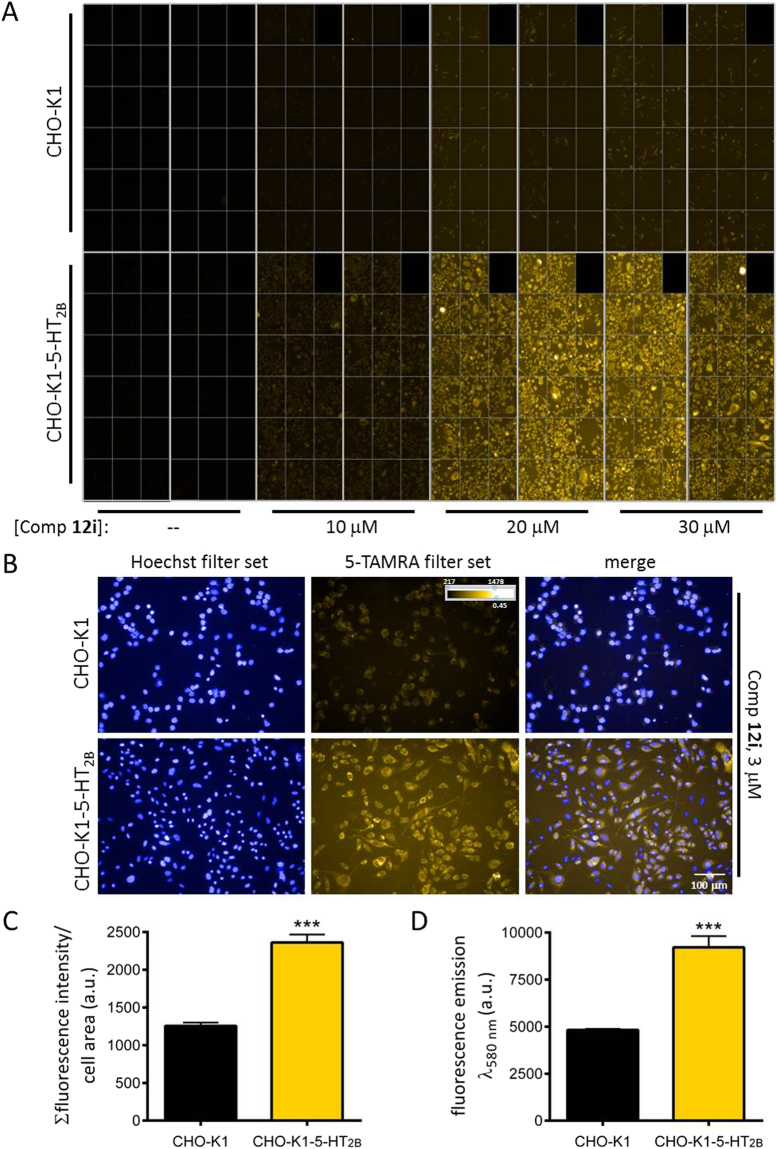
Labelling of 5-HT_2B_ receptors by compound 12i in cells. (**A**) Living parental untransfected CHO-K1 cells (CHO-K1) and CHO-K1 cells stably expressing 5-HT_2B_ receptors (CHO-K1-5-HT_2B_) were incubated in the absence or presence of the indicated concentrations of compound **12i** and, after compound removal, fluorescence images (excitation wavelength 520–550 nm, emission wavelength 560–630 nm, standard filter set for 5-TAMRA) were acquired using an automated high content imaging instrument at 20x magnification. (**B**) Sample images from CHO-K1 and CHO-K1-5-HT_2B_ cells labelled with 1 µg/mL Hoechst 33342 (for nuclear staining) and compound **12i** at a concentration of 3 μM, sampled from those quantified in (**C**,**D**). Minimum and maximum intensity and gamma correction of the images are shown in the colour scale in the panel. (C,D) Quantification of the fluorescence emission of compound **12i** in images from CHO-K1 and CHO-K1-5-HT_2B_ cells labelled with 1 µg/mL Hoechst 33342 (for nuclear staining) and compound **12i** at a concentration of 3 μM, both by image analysis (**C**) and by direct fluorescence measurement using a plate reader (**D**). The graphs show mean ± SEM of 4 wells, 5 fields/well (image analysis) and mean ± SEM of the same wells (plate reader). ***p < 0.001, two-way ANOVA and Bonferroni post tests.

While exhibiting a moderate 5-HT_2B_ affinity/selectivity profiles compound **12i** displayed a concentration-dependent specific labelling of CHO-K1-5-HT_2B_ cells and this resulted in brighter fluorescence images obtained from these cells in comparison with those from control CHO-K1 cells (Fig. 9). The best results were obtained for a 3 μM concentration of compound **12i**, which yielded the maximum window of specific labelling of the cells for the concentrations assayed (Fig. 9B). Under these conditions, the fluorescence intensity of the images collected from CHO-K1-5-HT_2B_ cell wells was clearly superior to that observed for control CHO-K1 cell wells (***p < 0.001, two-way ANOVA and Bonferroni posttests), as quantified both by automated image analysis and by direct measurement of fluorescence emission on a fluorescence plate reader (Fig. 9C,D). In the absence of compound, no substantial fluorescence signal was detected in the cells (Fig. 9A), which indicates a lack of signal specificity due to cell autofluorescence. These results support the specific binding of **12i** to 5-HT_2B_ receptors. Image acquisition at higher magnification (40x) and analysis retrieved similar quantitative results (Supplementary Figure S1). Furthermore, confocal microscopy imaging of CHO-K1-5-HT_2B_ cells labelled with compound **12i** in the same conditions also allowed visualization of the probe fluorescence, mainly located in intracellular compartments after 10 min incubation at 37 °C (Supplementary Figure S2).

The specificity and sensitivity of the **12i** probe towards 5-HT_2B_ receptors over other 5-HT_2_ receptor subtypes was evaluated by performing similar labelling experiments in CHO cells stably expressing 5-HT_2A_ receptors (CHO-FA4-5-HT_2A_). In this case, no statistically significant specific labelling was detected when compound **12i** was employed at the concentration of 3 μM (Supplementary Figure S3), and it was required to increase the **12i** concentration to 20 μM in order to detect certain specific labelling of 5-HT_2A_ receptors over background. However, even in these conditions (20 μM concentration of **12i**) the signal window over background achieved for 5-HT_2A_ labelling was considerably smaller than that detected in the case of 5-HT_2B_ labelling at a concentration of **12i** more than 6 times lower (3 μM) (Fig. 9 and Supplementary Figure S3). These results were in good agreement with the affinity values of compound **12i** obtained from our radioligand binding assays. Hence, compound **12i** allows direct visualization of 5-HT_2B_ receptors stably expressed in a cell line by live fluorescence microscopy imaging and constitutes a promising molecular probe for future studies.

## Conclusions

A set of fluorescent ligands based on the 1-(2,5-dimethoxy-4-iodophenyl)-propan-2-amine (DOI) chemotype has been developed. Some of the novel fluorescent probes (e.g. **12b**) show excellent affinity and selectivity profiles at the 5-HT_2B_ receptors, while retaining the agonistic functional behaviour of DOI. The study highlighted the most salient features of the structure-activity relationship in this series and these were supported by a molecular modelling study based on a receptor-driven docking model constructed on the basis of the crystal structure of the human 5-HT_2B_ receptor. One of the fluorescent ligands reported here enabled the visualization of 5-HT_2B_ receptors in live cells. Compound **12i** specifically labelled 5-HT_2B_ receptors stably expressed in CHO-K1 cells in a concentration-dependent manner in fluorescence microscopy studies. Hence, the probes described here are the first examples of 5-HT_2B_ selective fluorescent ligands and their availability should help to boost the GPCR biomolecular imaging field, thus enabling direct visualization and monitoring of spatiotemporal changes associated with 5-HT_2B_-related (patho)-physiological states. The ligand might be also useful for other assays where well characterized specific fluorescent probes and appropriate signal-to-background window are required, such as BRET-based binding assays.

## Methods

### Chemistry

Unless otherwise stated, all starting materials, reagents and solvents were purchased and used without further purification. The reactions were monitored by thin-layer chromatography (TLC) on 2.5 mm Merck silica gel GF 254 strips, and the purified compounds each showed a single spot; unless stated otherwise, UV light and/or iodine vapour were used to detect compounds. The purity and identity of all tested compounds were established by a combination of HPLC, elemental analysis, mass spectrometry and NMR spectroscopy as described below. Purification of isolated products was carried out by column chromatography (Kieselgel 0.040–0.063 mm, E. Merck) or medium pressure liquid chromatography (MPLC) on a CombiFlash Companion (Teledyne ISCO) with RediSep pre-packed normal-phase silica gel (35–60 µm) columns followed by recrystallization. Melting points were determined on a Gallenkamp melting point apparatus and are uncorrected. The NMR spectra were recorded on Bruker AM300 and XM500 spectrometers. Chemical shifts are given as δ values against tetramethylsilane as internal standard and *J* values are given in Hz. Mass spectra were obtained on a Varian MAT-711 instrument. High-resolution mass spectra were obtained on an Autospec Micromass spectrometer. Analytical HPLC was performed on an Agilent 1100 system using an Agilent Zorbax SB-Phenyl, 2.1 mm × 150 mm, 5 µm column with gradient elution using the mobile phases (A) H_2_O containing 0.1% CF_3_COOH and (B) MeCN and a flow rate of 1 mL/min. The purity of all tested compounds was determined to be >95%. The acid functionalized DOI precursors were synthesized by following previously described methods^44–48^. The experimental details of the synthesis of amines **8d**–**g** are reported in the supplementary information.

### General procedure for the synthesis of fluorescent amides 10 and 12

To a solution of the^44–48^ carboxylic acid (0.2 mmol) in anhydrous dichloromethane (10 mL) at 0 °C was added *N*,*N*-dicyclohexylcarbodiimide (0.25 mmol). To this mixture was slowly added the corresponding amine **8** (0.2 mmol) in anhydrous dichloromethane (5 mL) and the mixture was stirred at room temperature for 12 h. After completion of the reaction, as indicated by TLC, the mixture was diluted with water and extracted with dichloromethane. The organic phase was dried (Na_2_SO_4_), filtered and concentrated to afford the corresponding amide (**11**), which was used in the next step. To a solution of the amide (**11**) in dichloromethane (10 mL) at 0 °C was slowly added 4N HCl in dioxane (3 mL) and the mixture was stirred for 2 h at room temperature. After completion of the reaction, as indicated by TLC, the mixture was neutralized with saturated sodium bicarbonate, diluted with water and extracted with dichloromethane. The organic phase was dried over Na_2_SO_4_, filtered and concentrated to afford a residue that was purified by column chromatography on silica gel to afford the target amide.

### (±) 4-(2-(2-aminopropyl)-5-iodo-4-methoxyphenoxy)-*N*-(3-((5-(dimethyl-amino)naphthalene)-1-sulfonamido)propyl)butanamide (10a, 12a)

Green-yellow wax; 75% (102 mg); ^1^H NMR (300 MHz, CDCl_3_) δ (ppm): 8.50 (dt, *J* = 8.6, 0.9 Hz, 1H), 8.31 (dt, *J* = 8.7, 0.9 Hz, 1H), 8.17 (dt, *J* = 7.3, 1.0 Hz, 1H), 7.57–7.43 (m, 2H), 7.21–7.08 (m, 2H), 6.79 (t, *J* = 6.2 Hz, 1H), 6.61 (s, 1H), 3.85–3.71 (m, 6H), 3.29–3.11 (m, 3H), 2.99–2.74 (m, 9H), 2.60 (t, *J* = 6.8 Hz, 2H), 2.42–2.19 (m, 2H), 1.98 (t, *J* = 6.9 Hz, 2H), 1.51 (t, *J* = 5.6 Hz, 2H), 1.07 (d, *J* = 6.3 Hz, 3H). ^13^C NMR (75 MHz, CDCl_3_) δ (ppm): 173.2, 152.4, 151.9, 151.6, 135.2, 130.2, 129.8, 129.6, 129.3, 129.1, 128.3, 123.2, 122.6, 118.9, 115.2, 113.9, 82.8, 67.6, 57.1, 47.3, 45.4, 40.5, 40.1, 35.9, 32.8, 29.6, 25.3, 23.4. HRMS (ESI) *m*/*z*: calcd. for C_29_H_40_IN_4_O_5_S [M + H]^+^: 683.1759, found: 683.1760.

### (±) 7-(2-(2-aminopropyl)-5-iodo-4-methoxyphenoxy)-*N*-(3-((5-(dimethylami-no)naphthalene)-1-sulfonamido)propyl)heptanamide (10b)

Green-yellow wax, 71% (103 mg); ^1^H NMR (300 MHz, CDCl_3_) δ (ppm): 8.48 (dt, *J* = 8.5, 1.0 Hz, 1H), 8.33 (dt, *J* = 8.6, 0.9 Hz, 1H), 8.14 (dt, *J* = 7.3, 1.2 Hz, 1H), 7.57–7.41 (m, 2H), 7.22–7.04 (m, 2H), 6.88 (t, *J* = 5.9 Hz, 1H), 6.69 (s, 1H), 3.92–3.77 (m, 3H), 3.75–3.69 (m, 3H), 3.28–3.12 (m, 3H), 2.97–2.88 (m, 3H), 2.87–2.78 (m, 6H), 2.24–2.07 (m, 2H), 1.85–1.50 (m, 6H), 1.48–1.20 (m, 8H). ^13^C NMR (75 MHz, CDCl_3_) δ (ppm): 174.7, 156.1, 151.9, 144.6, 134.2, 133.6, 129.8, 129.7, 129.0, 128.9, 127.5, 124.5, 123.8, 122.3, 119.5, 114.7, 86.5, 68.7, 57.6, 46.2, 44.8, 40.0, 39.5, 38.6, 37.9, 29.7, 29.2, 28.6, 27.1, 26.2, 22.9. HRMS (ESI) *m*/*z*: calcd. for C_32_H_46_IN_4_O_5_S [M + H]^+^: 725.2228, found: 725.2216.

### (±) 11-(2-(2-aminopropyl)-5-iodo-4-methoxyphenoxy)-N-(3-((5-(dimethyl-amino)naphthalene)-1-sulfonamido)propyl)undecanamide (10c)

Green-yellow solid; mp: 171–173 °C, 63% (98 mg); ^1^H NMR (300 MHz, CDCl_3_) δ (ppm): 8.51 (dt, *J* = 8.5, 1.0 Hz, 1H), 8.32 (dt, *J* = 8.6, 0.9 Hz, 1H), 8.18 (dt, *J* = 7.4, 1.0 Hz, 1H), 7.69–7.38 (m, 2H), 7.23–7.07 (m, 2H), 6.67 (d, *J* = 8.8 Hz, 1H), 6.03 (t, *J* = 6.5 Hz, 1H), 3.94–3.82 (m, 2H), 3.76 (s, 3H), 3.41–3.30 (m, 1H), 3.24 (q, *J* = 6.3 Hz, 2H), 2.99–2.75 (m, 9H), 2.73–2.60 (m, 1H), 2.09 (t, *J* = 7.5 Hz, 2H), 1.72 (q, *J* = 7.0 Hz, 2H), 1.63–1.46 (m, 4H), 1.46–1.07 (m, 17H). ^13^C NMR (75 MHz, CDCl_3_) δ (ppm): 174.0, 152.3, 151.8, 135.3, 130.2, 129.8, 129.6, 129.0, 128.3, 128.1, 123.1, 122.5, 119.0, 115.2, 114.3, 83.1, 68.7, 57.1, 47.4, 45.4, 45.4, 39.9, 39.5, 36.5, 35.9, 29.6, 29.3, 29.1, 29.0, 29.0, 29.0, 26.0, 25.5, 21.7. HRMS (ESI) *m*/*z*: calcd. for C_36_H_54_IN_4_O_5_S [M + H]^+^: 781.2854, found: 781.2851.

### (±) 3-(2-(2-(2-(2-aminopropyl)-5-iodo-4-methoxyphenoxy)ethoxy)ethoxy)-N-(3-((5-(dimethylamino)naphthalene)-1-sulfonamido)propyl)propanamide (10d)

Green-yellow solid; mp: 107–109 °C, 57% (86 mg); ^1^H NMR (300 MHz, CDCl_3_) δ (ppm): 8.53 (dt, *J* = 8.5, 1.1 Hz, 1H), 8.30 (d, *J* = 8.7 Hz, 1H), 8.22 (dd, *J* = 7.3, 1.3 Hz, 1H), 7.56–7.47 (m, 2H), 7.21–7.11 (m, 2H), 6.83 (t, *J* = 5.2 Hz, 2H), 6.63 (s, 1H), 3.88 (td, *J* = 6.0, 2.9 Hz, 2H), 3.80 (s, 3H), 3.55 (t, *J* = 5.0 Hz, 2H), 3.51–3.34 (m, 9H), 3.09 (t, *J* = 5.0 Hz, 2H), 2.88 (s, 6H), 2.59 (t, *J* = 7.0 Hz, 2H), 2.39 (td, *J* = 7.2, 2.2 Hz, 2H), 2.09 (p, *J* = 6.7 Hz, 2H), 1.07 (d, *J* = 6.3 Hz, 3H). ^13^C NMR (75 MHz, CDCl_3_) δ (ppm): 173.6, 152.5, 151.8, 151.4, 134.8, 130.4, 129.8, 129.6, 129.2, 128.4, 125.9, 123.2, 122.5, 119.0, 115.3, 114.3, 83.9, 70.0, 69.9, 69.9, 69.3, 68.3, 57.1, 48.2, 45.4, 42.8, 39.2, 36.2, 33.2, 25.3, 18.1. HRMS (CI) *m*/*z*: calcd. for C_32_H_46_IN_4_O_7_S [M + H]^+^: 757.2132, found: 757.2161.

### (±) 4-(2-(2-aminopropyl)-5-iodo-4-methoxyphenoxy)-*N*-(3-((7-nitroben-zo[*c*][1,2,5]oxadiazol-4-yl)amino)propyl)butanamide (12b)

Orange solid; mp: 158–160 °C; 69% (84 mg); ^1^H NMR (300 MHz, CDCl_3_) δ (ppm): 8.30 (d, *J* = 8.8 Hz, 1H), 7.05 (s, 1H), 6.51 (s, 1H), 6.07 (d, *J* = 8.9 Hz, 1H), 3.79 (dt, J = 6.3, 4.7 Hz, 2H), 3.64 (s, 3H), 3.40 (dq, *J* = 8.6, 5.9 Hz, 2H), 3.28–3.16 (m, 3H), 2.90 (dd, *J* = 13.1, 5.5 Hz, 1H), 2.57 (dd, *J* = 13.1, 8.7 Hz, 1H), 2.38–2.26 (m, 2H), 2.01–1.89 (m, 2H), 1.87–1.75 (m, 2H), 1.19–0.94 (m, 5H). ^13^C NMR (75 MHz, CDCl_3_ + MeOD) δ (ppm): 174.5, 152.4, 151.3, 144.2, 137.2, 125.6, 122.3, 114.1, 98.4, 83.9, 67.9, 56.8, 41.0, 36.6, 36.5, 36.0, 32.9, 32.9, 29.4, 27.6, 25.3, 17.9. HRMS (CI) *m*/*z*: calcd. for C_23_H_30_IN_6_O_6_ [M + H]^+^: 613.1272, found: 613.1270.

### (±) 4-(2-(2-aminopropyl)-5-iodo-4-methoxyphenoxy)-*N*-(3-(1,3-dioxo-1,3-dihydro-2*H*-dibenzo[*e*,*g*]isoindol-2-yl)propyl)butanamide (12c)

Pale-yellow wax; 61% (83 mg); ^1^H NMR (300 MHz, CDCl_3_) δ (ppm): 9.21–8.94 (m, 2H), 8.72 (ddd, *J* = 8.7, 1.3, 0.8 Hz, 2H), 7.93–7.62 (m, 4H), 7.21 (q, *J* = 1.5 Hz, 1H), 6.81–6.56 (m, 2H), 4.05–3.84 (m, 3H), 3.77 (d, *J* = 0.7 Hz, 3H), 3.42–3.18 (m, 3H), 2.77–2.54 (m, 3H), 2.51–2.41 (m, 2H), 1.93 (dt, *J* = 13.0, 6.4 Hz, 4H), 1.18 (d, *J* = 6.3 Hz, 3H). ^13^C NMR (75 MHz, CDCl_3_) δ (ppm): 174.0, 171.9, 171.8, 155.1, 153.9, 132.2, 131.0, 128.6, 127.4, 127.3, 126.6, 125.4, 123.6, 123.1, 115.2, 86.3, 68.9, 58.2, 49.3, 40.4, 39.9, 37.4, 32.9, 26.9, 24.9, 22.8. HRMS (ESI) *m*/*z*: calcd. for C_33_H_35_IN_3_O_5_ [M + H]^+^: 680.1616, found: 680.1606.

### (±) 9-(3-(4-(2-(2-aminopropyl)-5-iodo-4-methoxyphenoxy)butanamido)pro-pyl)-10-methyl phenanthrene-9,10-dicarboxylate (12d)

Pale-yellow wax; 56% (79 mg); ^1^H NMR (300 MHz, CDCl_3_) δ (ppm): 8.84 (dt, *J* = 7.3, 1.8 Hz, 2H), 7.92 (dd, *J* = 7.5, 1.6 Hz, 1H), 7.87 (dd, *J* = 7.4, 1.6 Hz, 1H), 7.68 (td, *J* = 7.5, 1.6 Hz, 2H), 7.60 (tt, *J* = 7.5, 1.6 Hz, 2H), 7.15 (s, 1H), 6.64 (s, 1H), 6.58 (s, 1H), 4.22 (td, *J* = 12.2, 3.2 Hz, 2H), 4.04–3.86 (m, 5H), 3.81 (s, 3H), 3.60–3.42 (m, 3H), 3.00 (dd, *J* = 12.4, 6.9 Hz, 1H), 2.54 (dd, *J* = 12.6, 7.1 Hz, 1H), 2.35–2.23 (m, 2H), 2.03–1.92 (m, 2H), 1.90–1.80 (m, 2H), 1.70 (s, 2H), 1.05 (d, *J* = 5.9 Hz, 3H). ^13^C NMR (75 MHz, CDCl_3_) δ (ppm): 173.7, 168.9, 168.1, 152.4, 151.2, 130.9, 129.8, 129.4, 128.6, 127.7, 127.7, 127.4, 126.7, 126.5, 125.8, 122.9, 122.4, 122.3, 114.1, 83.8, 67.8, 64.3, 56.9, 52.8, 36.6, 32.9, 29.5, 28.1, 25.2, 22.5, 18.4. HRMS (CI) *m*/*z*: calcd. for C_34_H_38_IN_2_O_7_ [M + H]^+^: 713.1724, found: 713.1739.

### (±) 9-(3-(4-(2-(2-aminopropyl)-5-iodo-4-methoxyphenoxy)butanamido)pro-pyl) 10-methyl 2,3,6,7-tetrafluorophenanthrene-9,10-dicarboxylate (12e)

Pale-yellow wax; 52% (82 mg); ^1^H NMR (300 MHz, CDCl_3_) δ (ppm): 8.54–8.43 (m, 2H), 7.62 (d, *J* = 8.0 Hz, 1H), 7.52 (d, *J* = 8.1 Hz, 1H), 7.19 (s, 1H), 6.64 (s, 1H), 6.27 (s, 1H), 4.16 (td, *J* = 12.4, 1.9 Hz, 2H), 3.95 (s, 3H), 3.91–3.88 (m, 2H), 3.82 (s, 3H), 3.76 (td, *J* = 12.2, 3.6 Hz, 2H), 3.39–3.32 (m, 1H), 3.01 (dd, *J* = 12.1, 5.9 Hz, 1H), 2.68 (dd, *J* = 12.3, 6.1 Hz, 1H), 2.60–2.55 (m, 2H), 2.15–2.00 (m, 2H), 1.78–1.67 (m, 2H), 1.65 (s, 2H), 1.17 (d, *J* = 6.0 Hz, 3H). ^13^C NMR (75 MHz, CDCl_3_) δ (ppm): 174.6, 168.4, 167.8, 157.3, 154.8, 152.4, 151.7, 149.6, 148.7, 129.6, 128.3, 127.3, 127.0, 126.9, 125.6, 124.3, 123.9, 115.6, 114.4, 113.8, 86.5, 68.3, 63.6, 58.1, 52.3, 47.5, 39.8, 36.9, 32.3, 28.6, 24.3, 22.9. HRMS (ESI) *m*/*z*: calcd. for C_34_H_34_F_4_IN_2_O_7_ [M + H]^+^: 785.1341, found: 785.1341.

### (±) 5-(3-(4-(2-(2-aminopropyl)-5-iodo-4-methoxyphenoxy)butanamido)pro-pyl)-6-methyl-1,2,3,8,9,10-hexahydrodicyclopenta[b,h]phenanthrene-5,6-dicarbo-xylate (12f)

Pale-yellow wax; 63% (99 mg); ^1^H NMR (300 MHz, CDCl3) δ (ppm): 8.61–8.44 (m, 2H), 7.96–7.77 (m, 2H), 7.22–7.01 (m, 1H), 6.71–6.46 (m, 1H), 4.50 (q, *J* = 6.0 Hz, 2H), 4.00 (s, 3H), 3.90 (t, *J* = 6.1 Hz, 1H), 3.83–3.58 (m, 5H), 3.45–3.29 (m, 1H), 3.24–2.99 (m, 11H), 2.97–2.81 (m, 2H), 2.56–2.44 (m, 2H), 2.39–1.79 (m, 8H), 1.14 (d, *J* = 6.5 Hz, 3H). ^13^C NMR (75 MHz, CDCl_3_) δ (ppm): 172.9, 169.6, 168.7, 152.4, 151.4, 146.0, 145.8, 144.6, 144.4, 130.2, 127.5, 125.8, 125.6, 122.6, 122.3, 121.2, 121.1, 117.7, 114.2, 114.0, 83.3, 67.7, 63.6, 57.0, 52.6, 47.5, 38.6, 36.8, 33.2, 32.8, 31.6, 30.6, 28.3, 25.9, 24.7, 22.6. HRMS (ESI) *m*/*z*: calcd. for C_40_H_46_IN_2_O_7_ [M + H]^+^: 793.2344, found: 793.2347.

### (±) 6-(3-(4-(2-(2-aminopropyl)-5-iodo-4-methoxyphenoxy)butanamido)pro-pyl) 7-methyl pentaphene-6,7-dicarboxylate (12g)

Pale-yellow solid; mp: 150–152 °C; 64% (104 mg); ^1^H NMR (300 MHz, CDCl_3_) δ (ppm): 9.15 (d, *J* = 15.0 Hz, 2H), 8.46 (d, *J* = 24.9 Hz, 2H), 8.26–7.85 (m, 4H), 7.79–7.32 (m, 5H), 6.95 (s, 1H), 6.30 (s, 1H), 4.56 (t, *J* = 6.2 Hz, 2H), 4.06 (s, 3H), 3.80–3.28 (m, 8H), 3.14–2.83 (m, 1H), 2.61–2.45 (m, 1H), 2.38–2.25 (m, 2H), 2.14–1.81 (m, 4H), 1.30–1.10 (m, 5H). ^13^C NMR (75 MHz, CDCl_3_) δ (ppm): 173.5, 168.9, 168.1, 152.3, 151.0, 132.7, 132.6, 132.1, 130.6, 130.3, 128.5, 128.4, 128.3, 128.2, 128.1, 127.4, 126.7, 126.5, 125.5, 124.8, 124.7, 122.4, 122.3, 122.1, 113.8, 83.9, 68.0, 64.7, 56.8, 53.1, 48.2, 36.7, 36.2, 33.4, 28.3, 25.3, 18.1. HRMS (ESI) *m*/*z*: calcd. for C_42_H_42_IN_2_O_7_ [M + H]^+^: 813.2031, found: 813.2036.

### (±) 4-(2-(2-aminopropyl)-5-iodo-4-methoxyphenoxy)-*N*-(3-(1,3,8,10-tetraoxo-9-(tridecan-7-yl)-3,8,9,10-tetrahydroanthra[2,1,9-*def*:6,5,10-*d*′*e*′*f*′]diisoquinolin-2(1*H*)-yl)propyl)butanamide (12h)

Red solid, mp: 169–171 °C; 41% (82 mg); ^1^H NMR (300 MHz, CDCl_3_) δ (ppm): 8.44 (d, *J* = 7.7 Hz, 2H), 8.18 (d, *J* = 7.7 Hz, 2H), 8.12–8.05 (m, 4H), 7.48 (brs, 1H), 7.06 (s, 1H), 6.64 (s, 1H), 5.15 (t, *J* = 6.6 Hz, 1H), 4.18–4.09 (m, 1H), 4.01 – 3.80 (m, 4H), 3.78–3.71 (m, 1H), 3.65–3.52 (m, 5H), 3.36–3.23 (m, 2H), 3.12–3.04 (m, 1H), 2.93–2.84 (m, 1H), 2.55 (brs, 2H), 2.29–2.09 (m, 3H), 2.05–1.89 (m, 4H), 1.50–1.06 (m, 19H), 0.85 (t, *J* = 7.2 Hz, 6H). ^13^C NMR (75 MHz, CDCl_3_) δ (ppm): 175.5, 162.8, 162.7, 162.6, 162.6, 155.8, 154.1, 139.5, 139.1, 132.4, 131.8, 131.1, 127.5, 127.5, 126.8, 126.8, 124.9, 124.9, 124.6, 124.5, 124.4, 124.4, 124.4, 124.3, 124.3, 123.8, 123.8, 123.8, 123.8, 115.8, 87.3, 69.9, 57.1, 54.6, 48.4, 40.5, 38.9, 37.6, 35.7, 34.8, 34.8, 31.7, 31.7, 29.4, 29.4, 26.8, 26.6, 26.6, 25.7, 23.4 22.9, 22.9, 14.1, 14.1. HRMS (ESI) *m*/*z*: calcd. for C_54_H_62_IN_4_O_7_ [M + H]^+^: 1005.3657, found: 1005.3659.

### (±) *N*-(9-(2-((26-(2-(2-aminopropyl)-5-iodo-4-methoxyphenoxy)-3,7,23-trioxo-12,15,18-trioxa-4,8,22-triazahexacosyl)carbamoyl)phenyl)-6-(diethylamino)-3*H*-xanthen-3-ylidene)-*N*-ethylethanaminium (12i)

Dark red solid, mp: 151–153 °C, 35% (84 mg); ^1^H NMR (300 MHz, CDCl_3_) δ (ppm): 7.70–7.55 (m, 3H), 7.36–7.11 (m, 6H), 6.79–6.60 (m, 3H), 4.25–4.00 (m, 5H), 3.98–3.86 (m, 3H), 3.79 (s, 3H), 3.69–3.46 (m, 17H), 3.46–3.26 (m, 6H), 3.16–2.88 (m, 7H), 2.85–2.68 (m, 5H), 2.64–2.37 (m, 2H), 2.21–1.97 (m, 2H), 1.40–1.10 (m, 15H). ^13^C NMR (75 MHz, CDCl_3_) δ (ppm): 173.7, 173.4, 169.4, 169.0, 157.9, 157.8, 155.6, 152.4, 151.6, 136.1, 132.4, 132.2, 130.2, 129.7, 129.3, 128.1, 126.8, 122.2, 114.5, 113.9, 113.8, 113.7, 96.1, 83.6, 69.8, 69.5, 67.9, 63.1, 57.2, 52.9, 51.8, 51.7, 51.3, 50.5, 47.8, 46.0, 44.3, 39.2, 39.1, 38.7, 37.2, 35.3, 34.4, 30.6, 29.6, 24.9, 24.3, 17.8, 12.6. HRMS (ESI) *m*/*z*: calcd. for [C_57_H_78_IN_8_O_10_]^+^ [M]^+^: 1162.5080, found: 1162.5085.

### Biological methods

#### Cell Culture

Chinese hamster ovary K1 (CHO-K1) cells were maintained in Dulbecco’s modified Eagle’s medium-F12 (DMEM/F12) (GIBCO, Thermo Fisher Scientific, Spain) supplemented with 10% fetal bovine serum (FBS) (Sigma Aldrich, Spain), 2 mM L-glutamine (Sigma Aldrich, Spain) and 100 U/mL penicillin/0.1 mg/mL streptomycin (Sigma Aldrich, Spain). CHO cells stably expressing 5-HT_2A_ receptors (CHO-FA4-5-HT_2A_) were maintained in DMEM/F12 supplemented with 10% FBS, 2 mM L-glutamine and 100 U/mL penicillin/0.1 mg/mL streptomycin and 300 μg/mL hygromycin (Invitrogen, Thermo Fisher Scientific, Spain). CHO-K1 cells stably expressing the human 5-HT_2B_ receptor (CHO-K1-5-HT_2B_) were maintained in Advanced DMEM/F-12 (GIBCO, Thermo Fisher Scientific, Spain) supplemented with 1% FBS, 4 mM L-glutamine, 10 U/mL penicillin/0.01 mg/mL streptomycin and 0.4 mg/mL geneticin (G-418) (GIBCO, Thermo Fisher Scientific, Spain).

#### Radioligand competition binding assays at human 5-HT_2A_ receptors

Serotonin 5-HT_2A_ receptor competition binding experiments were carried out in membranes from CHO-FA4-5-HT_2A_ cells prepared in our group. On the day of the assay, membranes were defrosted and resuspended in binding buffer (50 mM Tris-HCl, pH 7.5). Each reaction well of a 96-well plate contained 80 μg of protein, 1 nM [^3^H]Ketanserin (50.3 Ci/mmol, Perkin-Elmer, Waltham, MA, USA), and different concentrations of the compounds in the range from 0.01 nM to 10 µM. Non-specific binding was determined in the presence of 1 μM methysergide (Sigma Aldrich, Spain). The reaction mixture was incubated at 37 °C for 30 min, after which samples were transferred to multiscreen GF/B 96-well plates (Millipore, Spain) pretreated with 0.5% polyethylenimine (PEI, Sigma Aldrich, Spain), filtered, and washed six times with 250 μL wash buffer (50 mM Tris-HCl, pH 6.6). The filters were dried, 35 μL Universol (MP Biomedicals, Spain) per well were added and radioactivity was detected in a microplate beta scintillation counter (Microbeta Trilux, Perkin-Elmer, Waltham, MA, USA).

#### Radioligand competition binding assays at human 5-HT_2B_ receptors

Serotonin 5-HT_2B_ receptor competition binding experiments were carried out in membranes from CHO-K1-5-HT_2B_ cells prepared in our group. On the day of the assay, membranes were defrosted and resuspended in binding buffer (50 mM Tris-HCl, 4 mM CaCl_2_, 0.1% ascorbic acid, pH 7.4). Each reaction well of a 96-well plate contained 5 μg of protein, 1 nM [^3^H]LSD (83.6 Ci/mmol, Perkin-Elmer, Waltham, MA, USA), and different concentrations of the compounds in the range from 0.01 nM to 10 µM. Non-specific binding was determined in the presence of 50 μM serotonin (Sigma Aldrich, Spain). The reaction mixture was incubated at 37 °C for 30 min, after which samples were transferred to multiscreen GF/C 96-well plates (Millipore, Spain) pretreated with 0.5% polyethylenimine (PEI, Sigma Aldrich, Spain), filtered, and washed four times with 250 μL wash buffer (50 mM Tris-HCl, pH 7.4). The filters were dried, 35 μL Universol (MP Biomedicals, Spain) per well were added and radioactivity was measured as described above.

#### Radioligand competition binding assays at human 5-HT_2C_ receptors

Serotonin 5-HT_2C_ receptor competition binding experiments were carried out in membranes from HeLa-5-HT_2C_ cells prepared in our group. On the day of assay, membranes were defrosted and resuspended in binding buffer (50 mM Tris-HCl, pH 7.5). Each reaction well of a 96-well plate contained 15 μg of protein, 4 nM [^3^H]Mesulergine (83.5 Ci/mmol, Perkin-Elmer, Waltham, MA, USA), and different concentrations of the compounds in the range from 0.01 nM to 10µM. Non-specific binding was determined in the presence of 10 μM mianserin (Sigma Aldrich, Spain). The reaction mixture was incubated at 27 °C for 1 h, after which samples were transferred to multiscreen GF/C 96-well plates (Millipore, Spain) pretreated with 0.5% polyethylenimine (PEI, Sigma Aldrich, Spain), filtered, and washed four times with 250 μL wash buffer (50 mM Tris-HCl, pH 6.6). The filters were dried, 35 μL Universol (MP Biomedicals, Spain) per well were added and radioactivity was measured as described above.

#### Functional Assays of IPs accumulation at human 5-HT_2B_ receptors

CHO-K1 cells stably expressing human 5-HT_2B_ receptor (CHO-K1-5-HT_2B_) cells were seeded into 96-well tissue culture plates at a density of 20000 cells/well. After 24 h, growth medium was replaced by serum-free medium containing 10 μCi/mL [^3^H]myo-inositol (20.3 Ci/mmol, Perkin-Elmer) for 24 h. After a labelling period of 24 h, cells were washed for 10 min at 37 °C with Hanks’ balanced salt solution (HBSS) supplemented with 20 mM HEPES, 20 mM LiCl and 2% fatty acid free bovine serum albumin (BSA) (assay buffer) and incubated in assay buffer in the absence (vehicle) or in the presence of the indicated concentrations of the compounds for 20 min at 37 °C. After the incubation time, assay buffer was discarded and 200 μL of 100 mM formic acid was added to the cells for 30 min at 4 °C and 20 μL of the lysate were transferred to a flexiplate plate (Perkin-Elmer, Waltham, MA, USA) together with 80 μL of a solution of RNA Binding YSi SPA Beads (Perkin-Elmer, Waltham, MA, USA) to measure accumulation of [^3^H]IPs (IP1,IP2, and IP3, collectively referred to as IPs). Radioactivity was quantified in a liquid scintillation counter WALLAC Microbeta TriLux 1450-023.

#### Data analysis of radioligand and functional assays

Concentration-response curves were fitted using Prism version 4.0 (GraphPad Software). A nonlinear regression fitting to a four parameter logistic equation and the Cheng–Prusoff equation were used to calculate EC_50_ values and *K*_i_, respectively.

#### Fluorescence microscopy imaging

For cell labelling studies, cells were plated onto 96-well plates (Cell Carrier; Perkin-Elmer, Waltham, MA, USA) pre-treated with poly-D-lysine hydrobromide 0.1 mg/mL (Sigma Aldrich, Spain) at a density of 10000 cells/well and cultured for 24 h. For cell labelling with compound **12i**, cultured media was removed and cells were washed with HBSS supplemented with 0.1% BSA and incubated in the same buffer for 30 min at 37 °C. The buffer was removed and cells were incubated in the absence or presence of the compound at the indicated concentrations in HBSS for 10 min at 37 °C. A 10^−2^ M stock solution of the compound was prepared in DMSO and then diluted to the final assay concentration in HBSS. Hoechst 33342 (1 µg/mL) (Thermo Fisher, Spain) was used to stain nuclei where indicated. After the incubation time, the supernatant was removed, cells were washed twice with HBSS and plates were subjected to microscopy. Fluorescence images were acquired using an Operetta high content imaging instrument (Perkin-Elmer, Waltham, MA, USA) at 20x and 40x magnification, at excitation wavelengths of 520–550 nm and emission wavelengths of 560–630 nm (standard 5-TAMRA filter set) for compound **12i**, and excitation wavelengths of 360–400 nm and emission wavelengths of 410–480 nm for Hoechst 33342 (standard Hoechst 33342 filter set).

#### Quantification of fluorescence intensity

The fluorescence intensity of the cells labelled with compound **12i** was quantified by image analysis of the acquired images using Harmony v3.5 software (Perkin-Elmer). Object identification was performed in the Hoechst channel followed by intensity measurement of the cell area of the objects in the 5-TAMRA channel and the results are expressed as the sum of the 5-TAMRA intensity in the cell region divided by the sum of cell area in each well. Five fields of view/well and 4 wells/condition were analyzed. Fluorescence intensity of the labeled cells was also quantified in the same plates and wells by measuring fluorescence emission (excitation wavelength 550 nm, emission wavelength 580 nm) on an Infinite M1000Pro fluorescence plate reader (TECAN, Männedorf, Switzerland). Data (mean ± SEM) obtained from both quantification methods were represented as bar graphs using Prism version 4.0 (GraphPad Software).

### In Silico Methods

#### Receptor preparation from the X-ray crystal structure

To simulate the interactions of 5-HT_2B_ with fluorescent probes, we used the recently crystallized structure of the receptor (PDB ID: 4IB4)^55^. The area of the structure, corresponding to the BRIL protein, was cut out. The protonation states were calculated at pH 7.4 using PROPKA^56^ software implemented in MOE (http://www.chemcomp.com/software.htm).

#### Ligand Docking

First, DOI was docked into the 5-HT2B receptor defining a centroid point in the residue Asp3.32 and expanding it to 20 Å around this residue using the GOLD software^57^. One hundred genetic algorithm runs were submitted and further scored employing the ASP scoring function. The ligands were restricted to form a salt bridge between their positively charged nitrogen and the carboxylate of Asp3.32. The best poses from this docking procedure were used as inputs to explore the conformational space of the ligands with the Low Mode Search function of MOE, which is a short MD simulation that uses velocities with little kinetic energy on the high-frequency vibrational modes, using the AMBER12:EHT force field, Born solvation, 300 K and default settings. The lowest energetic pose was then used as a starting point to link the dansyl probe using MOE. In order to explore stability and the conformational space, we carried out extended molecular dynamics (MD) simulations for the 5-HT_2B_ receptor in complex with DOI-linked fluorescence probes.

#### Extended MD simulations

The protein with the ligand docked was then embedded in a 1-palmitoyl-2-oleoyl-sn-glycero-3-phosphocholine bilayer using Charmm-gui^58^. Information obtained from the OPM database was employed to ensure proper alignment of the protein in membrane^59^. The system was solvated with TIP3 water. The ionic strength of the system was set to 0.15 M using NaCl ions. Parameters were derived from the Charmm36 forcefield. Missing parameters for the ligands were obtained from CGenFF^60^. Simulations were carried out in ACEMD^61^ and the hydrogen mass-repartitioning scheme employed in ACEMD allowed us to use a 4 fs timestep^62^. The system was minimized for 3000 steps, and then subjected to 2 ns of NPT equilibration at a constant pressure of 1.01325 bar to ensure proper lipid packing in the membrane. Afterwards we proceeded with NVT simulations at a temperature of 300 K. For each complex (complex 1: 10a-5-HT_2B_ and complex 2: 10b-5-HT_2B_) we generated 4 individually built starting structures and simulated them for 200 ns each, yielding a total time of 800 ns per complex.

## Electronic supplementary material

Supplementary Information
