# Supplementary material for: Development of Fluorescent Probes that Target Serotonin 5-HT2B Receptors
Source: Sci Rep. 2017 Sep 7;7:10765. doi: 10.1038/s41598-017-11370-2 (PMC5589878; doi:10.1038/s41598-017-11370-2)
Supplement: Supplementary file 1 — Supplementary Information [file 41598_2017_11370_MOESM1_ESM.docx]

**SUPLEMENTARY INFORMATION**

**Development of Fluorescent Probes that Target**

**Serotonin 5-HT_2B_ Receptors**

Jhonny Azuaje,^1,2,3^ Paula López,^1,2,3^ Alba Iglesias,^3,4^ Rocío A. de la Fuente,^3,4^ José M. Pérez-Rubio,^1,2^ Diego García,^1^ Tomek Maciej Stępniewski,^5,6^ Xerardo García-Mera,^2,3^ José M. Brea,^3,4^ Jana Selent,^5,6^ Dolores Pérez^1,2^ Marián Castro,^3,4^ María I. Loza,^3,4^

and Eddy Sotelo^1,2,3*^

^1^Centro Singular de Investigación en Química Biolóxica e Materiais Moleculares (CIQUS), Universidade de Santiago de Compostela, E-15782 Santiago de Compostela, Spain. ^2^Departamento de Química Orgánica, Facultad de Farmacia, Universidade de Santiago de Compostela, E-15782 Santiago de Compostela, Spain. ^3^Instituto de Farmacia Industrial (IFI), Universidade de Santiago de Compostela, E-15782 Santiago de Compostela, Spain. ^4^Centro Singular de Investigación en Medicina Molecular e Enfermidades Crónicas (CIMUS), Universidade de Santiago de Compostela, E-15782 Santiago de Compostela, Spain. ^5^PharmacoInformatics Group, Research Program on Biomedical Informatics (GRIB) PRBB, Barcelona, 08003, Spain, ^6^Faculty of Chemistry, Biological and Chemical Research Centre, University of Warsaw, 02-093 Warsaw, Poland.

**SUPPLEMENTARY INFORMATION**

**- Experimental data for the synthesis of amines**

**- Confocal microscopy imaging studies.**

**-Supplementary Figure Legends**

**-Supplementary Figure S1. Imaging and quantification of the labelling of 5-HT_2B_ receptors by compound 12i in cells at 40× image acquisition magnification.**

**-Supplementary Figure S2. Imaging of the labelling of 5-HT_2B_ receptors by compound 12i in cells by confocal fluorescence microscopy at 63× magnification.**

**-Supplementary Figure S3. Cell imaging studies with compound 12i at 5-HT_2A_ receptors performed in the same conditions as those employed for 5-HT_2B_ receptors.**

**- References**

**Experimental data for the synthesis of amines**

**Synthesis of** **N-Boc-protected 2-(2-aminoethyl)-1*H*-dibenzo[*e*,*g*]isoindole-1,3(2*H*)-dione 8c:^1^** A solution of dimethyl phenanthrene-9,10-dicarboxylate (80.0 mg, 0.27 mmol) and *tert*-butyl (3-aminopropyl)carbamate^1^ (70.0 mg, 0.32 mmol) in dry pyridine (5.4 mL) was heated at 105 °C for 24 h. The solvent was evaporated under reduced pressure and the residue was chromatographed (SiO_2_, AcOEt/CH_2_Cl_2_ 1:9) to afford the N-Boc-protected derivative of imide **8c** as a yellow solid, 80% (96 mg); ^1^H RMN (250 MHz, CDCl_3_) δ (ppm): 9.10 (dd, *J* = 7.0, 2.3 Hz, 2H), 8.69 (dd, *J* = 7.4, 2.0 Hz, 2H), 7.86–7.68 (m, 4H), 4.55 (s, 1H), 3.74 (t, *J* = 7.2 Hz, 2H), 3.11 (dd, *J* = 12.0, 5.9 Hz, 2H), 1.84–1.59 (m, 4H), 1.43 (s, 9H), 1.56–1.32 (m, 4H); ^13^C NMR (63 MHz, CDCl_3_) δ (ppm): 170.1 (2C), 156.1 (C), 133.4 (2C), 129.5 (2CH), 128.5 (2CH), 127.5 (2C), 126.3 (2CH), 125.6 (2C), 123.2 (2CH), 79.3 (C), 37.8 (CH_2_), 30.0 (CH_2_), 29.8 (CH_2_), 28.8(CH_2_), 28.5(3CH_3_), 26.6(CH_2_), 26.5 (CH_2_). MS (EI), *m/z* (%): 404 (M^+^, 17), 348 (91), 260 (100); HRMS (EI) C_24_H_24_N_2_O_4_, calculated: 404.1736, found: 404.1723. UV/Vis (CHCl_3_), λ_max_ (ε, mol^-1^ dm^3^ cm^-1^): 382 (5382), 293 (14530), 282 (22970) nm.

**Synthesis N-Boc derivatives of amines 8d-8g**: **1. General procedure for the synthesis of dimethyl phenanthrene-9,10-dicarboxylates.** To a mixture of the corresponding *o*-(trimethylsilyl)aryl triflate^2^ (0.3 mmol), dimethyl acetylene-dicarboxylate (DMAD, 0.45 mmol) and Pd(PPh_3_)_4_ (0.03 mmol) in dry acetonitrile (6 mL), finely powdered anhydrous CsF was added. The mixture was stirred under argon at rt for 12 h, then H_2_O (10 mL) was added, the phases were separated and the organic layer was dried on anhydrous Na_2_SO_4_ and concentrated under reduce pressure. The crude residue was chromatographed (SiO_2_, CH_2_Cl_2_/hexanes).

**Dimethyl phenanthrene-9,10-dicarboxylate**:^3^ white solid, 84% (38 mg); ^1^H RMN (300 MHz, CDCl3) δ (ppm): 8.67 (d, *J* = 7.9 Hz, 2H), 8.15 (dd, *J* = 8.1, 1.1 Hz, 2H), 7.71–7.74 (m, 4H), 4.03 (s, 6H) ppm.

**Dimethyl 2,3,6,7-tetrafluorophenanthrene-9,10-dicarboxylate**:^3^ white solid, 64% (35 mg); ^1^H RMN (300 MHz, CDCl3) δ (ppm): 8.12 (dd, *J* = 11.6, 7.5 Hz, 2H), 7.93 (dd, *J* = 11.7, 8.0 Hz, 2H), 3.97 (s, 6H) ppm.

**Dimethyl 1,2,3,8,9,10-hexahydrodicyclopenta[*b,h*]phenanthrene-9,10-dicarboxy-late**: white solid, m.p. 266-268 ºC, 55% (30 mg); ^1^H RMN (500 MHz, CDCl3) δ (ppm): 8.53 (s, 2H), 7.93 (s, 2H), 4.02 (s, 6H), 3.15 (t, *J* = 7.4 Hz, 2H), 3.10 (t, *J* = 7.4 Hz, 2H), 2.19 (p, *J* = 7.4 Hz, 2H); ^13^C NMR (126 MHz, CDCl3) δ (ppm): 169.2 (2C), 145.9 (2C), 144.6 (2C), 130.5 (2C), 128.9 (2C), 125.9 (2C), 121.5 (2CH), 117.8 (2CH), 52.8 (2CH_3_), 33.3 (2CH_2_), 32.9 (2CH_2_), 26.1 (2CH_2_). MS (EI), *m/z* (%): 374 (M^+^, 100), 343 (62); HRMS (EI) C_24_H_22_O_4_, calculated: 374.1518, found: 374.1511.

**Dimethyl pentaphene-6,7-dicarboxylate**: yellow solid, m.p. 240-242ºC, 60% (36 mg); ^1^H RMN (300 MHz, CDCl_3_) δ (ppm): 9.18 (s, 2H), 8.52 (s, 2H), 8.10 (d, *J* = 8.1 Hz, 2H), 7.99 (d, *J* = 7.6 Hz, 2H), 7.68–7.48 (m, 4H), 4.11 (s, 6H); ^13^C NMR (75 MHz, CDCl3) δ (ppm): 168.4 (2C), 132.9 (2C), 132.4 (2C), 130.8 (2C), 128.9 (2C), 128.6 (2CH), 128.3 (2CH), 127.3 (2CH), 126.9 (2CH), 126.6 (2CH), 125.4 (2C), 122.3 (2CH), 52.8 (2CH_3_). MS (EI), *m/z* (%): 394 (M^+^, 100); HRMS (EI) C_26_H_18_O_4_, calculated: 394.1205, found: 394.1211.

***General procedure for the synthesis of N-Boc protected amines 8d-g:*** A freshly prepared solution of LDA (≈ 0.6 mmol) in THF (1 mL) was cooled to -78 °C and transferred, *via* cannula, to a solution of *tert*-butyl (3-hydroxypropyl)carbamate^4^ (≈ 0.5 mmol) in THF (1.1 mL). The stirred mixture was allowed to reach room temperature for 1 h. To the resulting alkoxide suspension, a solution of the corresponding dimethyl phenanthrene-9,10-dicarboxylate (or pentaphene-6,7-dicarboxylate, for **8g**) (≈ 0.25 mmol) in THF (2.5 mL) was dropwise added *via* cannula, and stirring at room temperature was kept for 12 h. The solvent was evaporated under reduced pressure and the residue was chromatographed (SiO_2_, AcOEt/CH_2_Cl_2_ 1:19) to afford the corresponding *N*-Boc-protected amine.

***N*-Boc-protected 9-(3-aminopropyl) 10-methyl phenanthrene-9,10-dicarboxylate 8d.** Following the general procedure described above, LDA (0.75 mmol) was reacted with *tert*-butyl (3-hydroxypropyl)carbamate (119 mg, 0.68 mmol) and dimethyl phenanthrene-9,10-dicarboxylate (100 mg, 0.34 mmol) to afford the N-Boc-protected derivative of amine **8d** as a white solid, 52% (77 mg); ^1^H RMN (500 MHz, CDCl_3_) δ (ppm): 8.72 (d, *J* = 8.3 Hz, 2H), 8.15 (dd, *J* = 3.5, 0.9 Hz, 1H), 8.13 (dd, *J* = 3.4, 0.9 Hz, 1H), 7.75 (td, *J* = 7.3, 1.9 Hz, 2H), 7.67 (td, *J* = 8.1, 3.6 Hz, 2H), 4.86 (s, 1H), 4.52 (t, *J* = 6.3 Hz, 2H), 4.04 (s, 3H), 3.29 (d, *J* = 6.2 Hz, 2H), 2.01 (p, *J* = 6.4 Hz, 2H), 1.42 (s, 9H); ^13^C NMR (126 MHz, CDCl_3_) δ (ppm): 168.6 (C), 168.2 (C), 156.1 (C), 131.2 (C), 131.1 (C), 130.3 (C), 129.7 (C), 128.7 (CH), 128.6 (CH), 127.9 (CH), 127.8 (CH), 127.2 (C), 127.1 (C), 126.9 (CH), 126.9 (CH), 123.04 (CH), 123.02 (CH), 79.4 (C), 63.7 (CH_2_), 52.9 (CH_3_), 37.5 (CH_2_), 29.0 (CH_2_), 28.5 (3CH_3_). MS (EI), *m/z* (%): 437 (M^+^, 8), 263 (100); HRMS (EI) C_25_H_27_NO_6_, calculated: 437.1838, found: 437.1838.

***N*-Boc-protected 9-(3-aminopropyl) 10-methyl 2,3,6,7-tetrafluorophenanthrene-9,10-dicarboxylate 8e:** Following the general procedure described above, LDA (0.53 mmol) was reacted with *tert*-butyl (3-hydroxypropyl)carbamate (84.0 mg, 0.48 mmol) and dimethyl 2,3,6,7-tetrafluorophenanthrene-9,10-dicarboxylate (90 mg, 0.24 mmol) to afford the N-Boc-protected derivative of amine **8e** as a white solid, 50% (61 mg); ^1^H RMN (500 MHz, CDCl_3_) δ (ppm): 8.24 (dd, *J* = 11.4, 7.7 Hz, 2H), 8.07–7.98 (m, 2H), 4.76 (s, 1H), 4.50 (t, *J* = 6.3 Hz, 2H), 4.04 (s, 3H), 3.27 (d, *J* = 6.1 Hz, 2H), 2.05–1.94 (m, 2H), 1.43 (s, 9H); ^13^C NMR (126 MHz, CDCl_3_) δ (ppm): 167.5 (C), 167.2 (C), 156.1 (C), 152.5 (C), 151.9 (C), 151.7 (C), 150.5 (C), 149.9 (C), 149.7 (C), 129.9 (C), 129.2 (C), 127.9 (C), 124.4 (C), 114.7 (2CH), 111.0 (2CH), 79.6 (C), 64.2 (CH_2_), 53.3 (CH_3_), 37.4 (CH_2_), 29.2 (CH_2_), 28.5 (3CH_3_). MS (EI), *m/z* (%): 509 (M^+^, 0.3), 335 (100); HRMS (EI) C_25_H_23_NO_6_F_4_, calculated: 509.1462, found: 509.1476.

***N*-Boc-protected 5-(3-aminopropyl) 6-methyl 1,2,3,8,9,10-hexahydrodicyclo-penta[*b*,*h*]phenanthrene-5,6-dicarboxylate 8f:** Following the general procedure described above, LDA (0.62 mmol) was reacted with *tert*-butyl (3-hydroxypropyl)carbamate (98.0 mg, 0.56 mmol) and dimethyl 1,2,3,8,9,10-hexahydro-dicyclopenta[*b,h*]phenanthrene-9,10-dicarboxylate (105 mg, 0.28 mmol) to afford the N-Boc-protected derivative of amine **8f** as a white solid, 46% (66 mg); ^1^H RMN (300 MHz, CDCl_3_) δ (ppm): 8.50 (s, 2H), 7.90 (s, 2H), 4.93 (s, 1H), 4.50 (t, *J* = 6.3 Hz, 2H), 4.02 (s, 3H), 3.28 (q, *J* = 6.2 Hz, 2H), 3.17–3.00 (m, 8H), 2.17 (p, *J* = 7.1 Hz, 4H), 2.06–1.92 (m, 2H), 1.43 (s, 9H); ^13^C NMR (75 MHz, CDCl_3_) δ (ppm): 169.2 (C), 168.8 (C), 156.1 (C), 145.9 (C), 145.8 (C), 144.47 (C), 144.45 (C), 130.4 (C), 130.3 (C), 129.1 (C), 128.6 (C), 125.85 (C), 125.81 (C), 121.3 (2CH), 117.8 (2CH), 79.2 (C), 63.3 (CH_2_), 52.7 (CH_3_), 37.5 (CH_2_), 33.2 (2CH_2_), 32.9 (2CH_2_), 28.9 (CH_2_), 28.4 (3CH_3_), 25.9 (2CH_2_). MS (EI), *m/z* (%): 517 (M^+^, 11), 343 (100).

**N-Boc-protected 6-(3-aminopropyl) 7-methyl pentaphene-6,7-dicarboxylate 8g:** Following the general procedure described above, LDA (0.70 mmol) was reacted with *tert*-butyl (3-hydroxypropyl)carbamate (98.0 mg, 0.56 mmol) and dimethyl pentaphene-6,7-dicarboxylate (125 mg, 0.32 mmol) to afford the *N*-Boc-protected derivative of amine **8g** as a yellow solid, 58% (99 mg); ^1^H RMN (300 MHz, CDCl_3_) δ (ppm): 9.09 (s, 2H), 8.42 (d, *J* = 6.8 Hz, 2H), 8.06 (d, *J* = 8.0 Hz, 2H), 7.92 (t, *J* = 7.4 Hz, 2H), 7.57 (dd, *J* = 13.9, 6.0 Hz, 2H), 7.52 (dd, *J* = 13.6, 5.5 Hz, 2H), 4.93 (s, 1H), 4.60 (t, *J* = 6.2 Hz, 2H), 4.11 (s, 3H), 3.41–3.28 (m, 2H), 2.13–2.01 (m, 2H), 1.44 (s, 9H); ^13^C NMR (75 MHz, CDCl_3_) δ (ppm): 168.5 (C), 168.1 (C), 156.1 (C), 132.8 (2C), 132.7 (2C), 132.2 (2C), 130.9 (C), 130.4 (C), 128.5 (2CH), 128.2 (2CH), 127.25 (2CH), 126.7 (2CH), 126.5 (2CH), 125.1 (2C), 122.2 (2CH), 79.4 (C), 63.7 (CH_2_), 52.9 (CH_3_), 37.6 (CH_2_), 29.2 (CH_2_), 28.5 (3CH_3_). MS (EI), *m/z* (%): 537 (M^+^, 91), 380 (29), 348 (100).

**Synthesis of** ***N*-Boc-protected *N-*(3-aminopropyl)-*N-*(1-hexylheptyl)perylene-3,4,9,10-tetracarboxylic diimide 8h:** A solution of *N*-(1-hexylheptyl)perylene-3,4,9,10-tetracarboxyl-3,4-anhydride-9,10-imide^5^ (130 mg, 0.22 mmol) and *tert*-butyl (3-aminopropyl)carbamate^4^ (84.2 mg, 0.48 mmol) in dry toluene (4.4 mL) was heated at 120 °C for 12 h. The solvent was evaporated under reduced pressure and the residue was chromatographed (SiO_2_, AcOEt/CH_3_Cl 1:19) to afford the N-Boc-protected derivative of diimide **8h** as a red solid, 78% (125 mg); ^1^H RMN (500 MHz, CDCl_3_) δ (ppm): 8.60 (m, *J* = 8.0 Hz, 4H), 8.54 (d, *J* = 8.1 Hz, 2H), 8.52 (d, *J* = 8.1 Hz, 2H), 5.24 (s, 1H), 5.18 (tt, *J* = 9.3, 5.9 Hz, 1H), 4.29 (t, *J* = 6.5 Hz, 2H), 3.20 (d, *J* = 5.9 Hz, 2H), 2.30–2.19 (m, 2H), 1.97 (p, *J* = 6.2 Hz, 2H), 1.92–1.83 (m, 2H), 1.46 (s, 9H), 1.39–1.18 (m, 16H), 0.83 (t, *J* = 6.9 Hz, 6H); ^13^C NMR (126 MHz, CDCl_3_) δ (ppm): 167.5 (C), 167.2 (C), 156.1 (C), 152.5 (C), 151.9 (C), 151.7 (C), 150.5 (C), 149.9 (C), 149.7 (C), 129.9 (C), 129.2 (C), 127.9 (C), 124.4 (C), 114.7 (2CH), 111.0 (2CH), 79.6 (C), 64.2 (CH_2_), 53.3 (CH_3_), 37.4 (CH_2_), 29.2 (CH_2_), 28.5 (3CH_3_). MS (MALDI-TOF), *m/z* (%): 729.285 (M^+^, 5), 728.283 (M^+^-1, 16), 630.239 (100).

**Confocal microscopy imaging studies.** For confocal microscopy studies, cells were labelled following the same protocol as for fluorescence microscopy imaging studies. Cells were plated onto 96-well glass bottom plates poly-d-lysine coated (MatTek Corporation, Ashland, MA, USA) at a density of 10000 cells/well and cultured for 36 h. For cell labelling with compound **12i**, cultured media was removed and cells were washed with HBSS supplemented with 0.1% BSA and incubated in the same buffer for 30 min at 37 ºC. The buffer was removed and cells were incubated in the absence or presence of compound **12i** at the concentration of 3 µM in HBSS for 10 min at 37 ºC. Hoechst 33342 (1 µg/mL) (Thermo Fisher, Spain) was used to stain nuclei. After the incubation time, the supernatant was removed, cells were washed twice with HBSS and plates were subjected to confocal microscopy using a Leica TCS SP5 confocal laser scanning microscope equipped with a white laser and a 63× oil immersion objective, at excitation wavelength of 560 nm and emission wavelengths of 580–697 nm for compound **12i**, and using a 405 diode laser for Hoechst 33342.

**SUPPLEMENTARY FIGURE LEGENDS**

**Supplementary Figure S1. Imaging and quantification of the labelling of 5-HT_2B_ receptors by compound 12i in cells at 40× image acquisition magnification.** A) Living parental untransfected CHO-K1 cells (CHO-K1) and CHO-K1 cells stably expressing 5-HT_2B_ receptors (CHO-K1-5-HT_2B_) were incubated in the absence or presence of different concentrations of compound **12i** and, after compound removal, fluorescence images (excitation wavelength 520–550 nm, emission wavelength 560–630 nm, standard filter set for 5-TAMRA) were acquired using an automated high content imaging instrument at 40× magnification. The panel shows images (2 wells per cell type, 5 fields per well) corresponding to the incubation with compound **12i** at the concentration of 3 µM. B) Quantification by image analysis of the fluorescence emission of compound **12i** in 40× magnification images from CHO-K1 and CHO-K1-5-HT_2B_ cells labelled with 1 µg/mL Hoechst 33342 (for nuclear staining) and 3 µM compound **12i**. The graphs show mean ± SEM of 3 wells, 5 (CHO-K1) or 9 (CHO-K1-5-HT_2B_) fields/well. ***p < 0.001, two-way ANOVA and Bonferroni posttests. C) Sample images from CHO-K1 and CHO-K1-5-HT_2B_ cells labelled with 1 µg/mL Hoechst 33342 (for nuclear staining) and 3 µM compound **12i**, sampled from those quantified in (B). Minimum and maximum intensity and gamma correction of the images are shown in the colour scale in the panel.

**Supplementary Figure S2. Imaging of the labelling of 5-HT_2B_ receptors by compound 12i in cells by confocal fluorescence microscopy at 63× magnification.** A) Sample fields of living CHO-K1 and CHO-K1-5-HT_2B_ cells labelled with 1 µg/mL Hoechst 33342 and 3 µM compound **12i**. Cells were incubated with the labelling compounds for 10 min at 37ºC and compounds were washed out prior to image acquisition. Images (405 diode laser for Hoechst 33342, excitation wavelength 560 nm with white laser and emission wavelength 580–697 nm for compound **12i**) were acquired using a Leica TCS SP5 confocal laser scanning microscope equipped with a 63× oil immersion objective. The panel shows an individual bright field plane and the maximum projections of the two fluorescence channels as well as their overlay image. B) A z-scan series of living CHO-K1-5-HT_2B_ cells labelled with 1 µg/mL Hoechst 33342 and 3 µM compound **12i** as previously described. Images were acquired in the same conditions as in panel A).

**Supplementary Figure S3. Cell imaging studies with compound 12i at 5-HT_2A_ receptors performed in the same conditions as those employed for 5-HT_2B_ receptors.** A) Living parental untransfected CHO-K1 cells (CHO-K1) and CHO cells stably expressing 5-HT_2A_ receptors (CHO-FA4-5-HT_2A_) were incubated in the absence or presence of different concentrations of compound **12i** and, after compound removal, fluorescence images (excitation wavelength 520–550 nm, emission wavelength 560–630 nm, standard filter set for 5-TAMRA) were acquired using an automated high content imaging instrument at 20× magnification. The panel shows sample images (1 well, 4 fields/well) from CHO-K1 and CHO-FA4-5-HT_2A_ cells labelled with 1 µg/mL Hoechst 33342 (for nuclear staining) and compound **12i** at the indicated concentrations, sampled from those quantified in (B, C). Minimum and maximum intensity and gamma correction of the images are shown in the colour scale in the panel. B, C) Quantification of the fluorescence emission of compound 12i in images from CHO-K1 and CHO-FA4-5-HT_2A_ cells labelled with 1 µg/mL Hoechst 33342 (for nuclear staining) and compound 12i at the indicated concentrations, both by image analysis (B) and by direct fluorescence measurement using a plate reader (C). No significant labelling of 5-HT_2A_ receptors by compound **12i** at the concentration of 3 µM was detected. The graphs show mean ± SEM of 3-4 wells, 5 fields/well (image analysis) and mean ± SEM of the same wells (plate reader). *p < 0.05, ***p < 0.001, two-way ANOVA and Bonferroni posttests.

**Supplementary Figure S1**


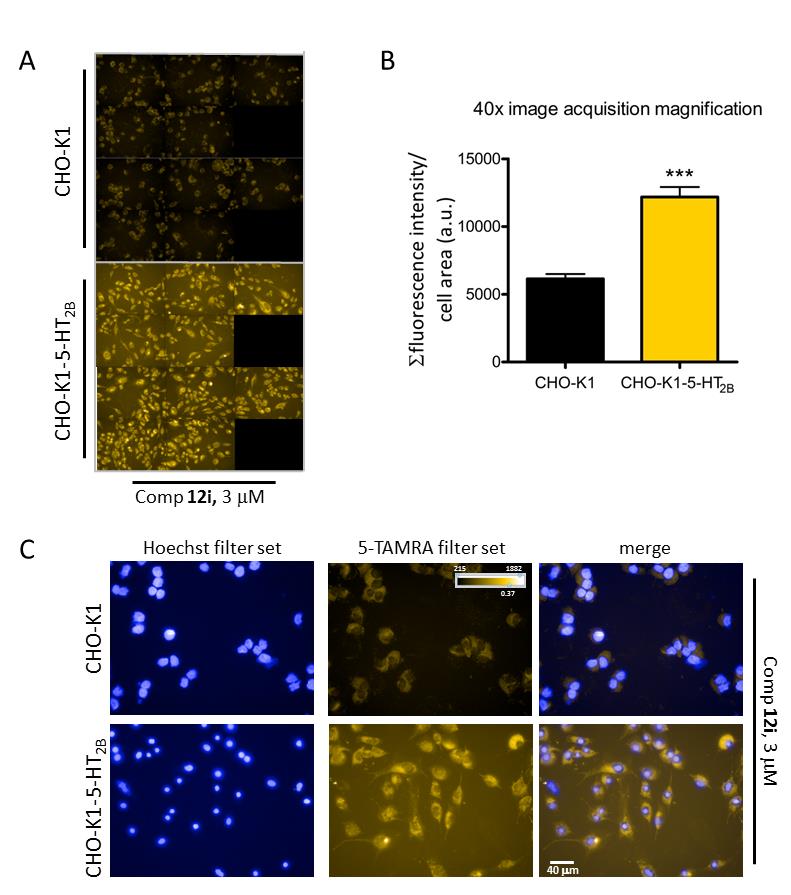


**Supplementary Figure S2**
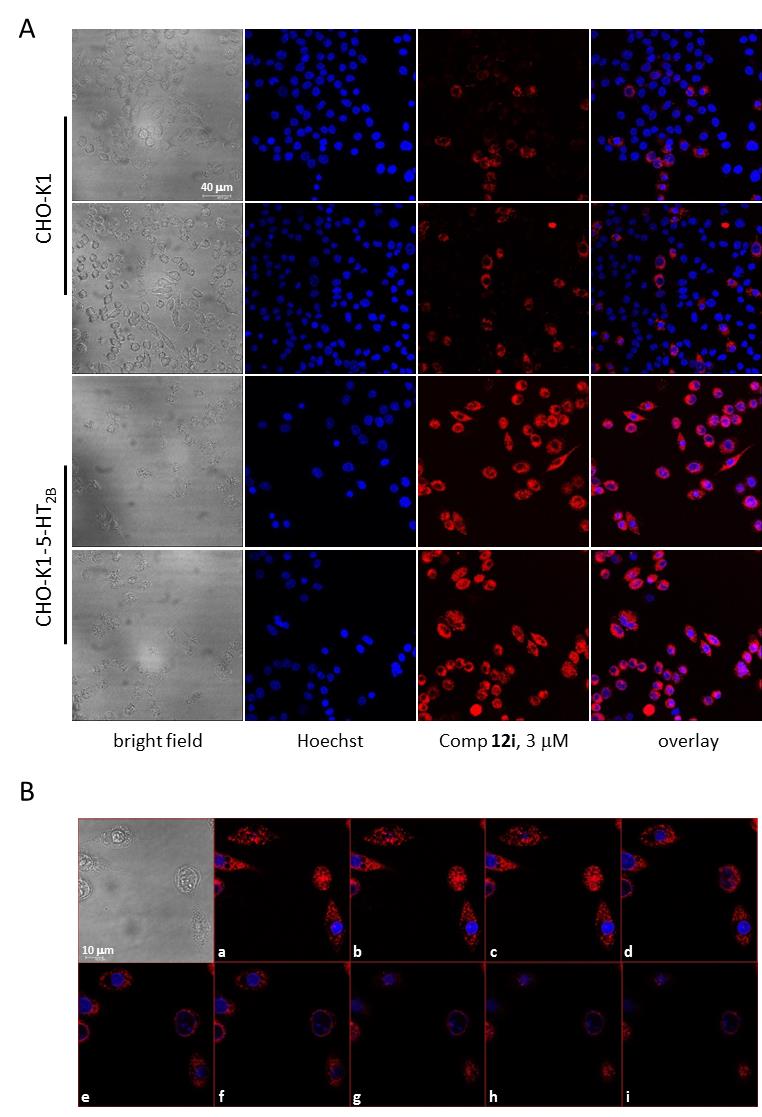


**Supplementary Figure S3**


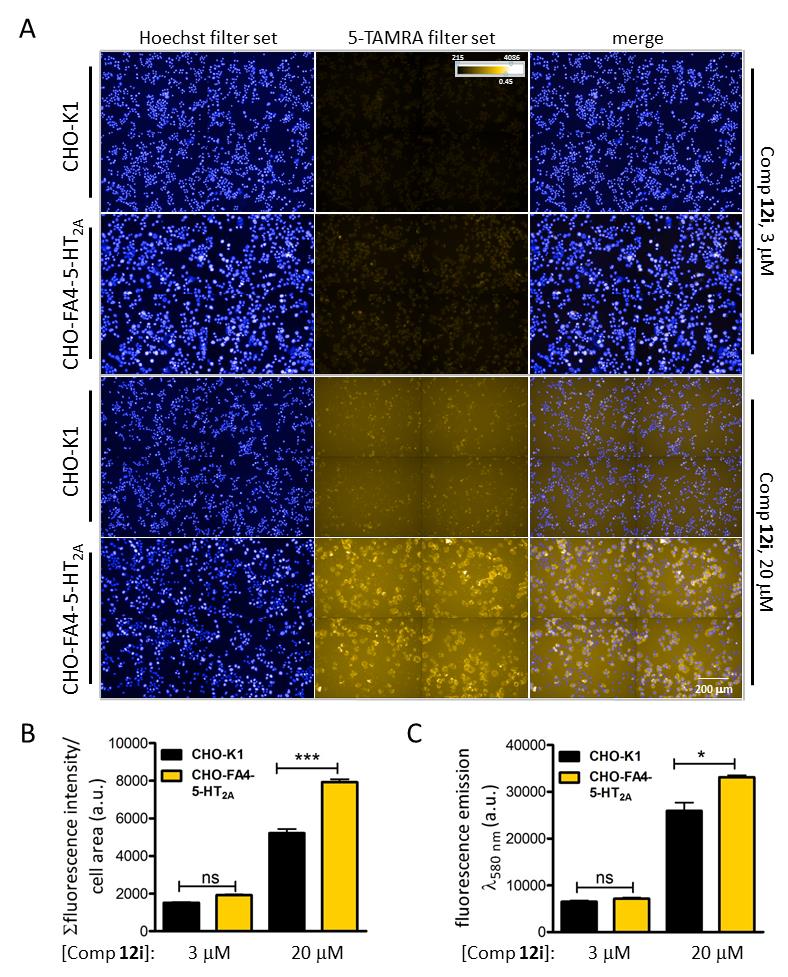


**References:**

1. Krapcho, A. P.; Kuell, S. C. Mono-Protected diamines. *N*-*tert-*butoxycarbonyl-α,ω-alkanediamines from α,ω-alkanediamines. *Synth. Commun.* **20**, 2559-2564 (1990**)**.
2. (a) Peña, D.; Cobas, A.; Pérez, D.; Guitián, E. *Synthesis* **10**, 1454-1458 (2002). (b) Hermann, K.; Pratumyot, Y.; Polen, S.; Hardin, A. M.; Dalkilic, E.; Dastan, A.; Badjic, J. D. *Chem.* *Eur. J.* **9**, 3550-3555 (2015).
3. Peña, D., Pérez, D., Guitián, E., Castedo, L. Palladium-catalyzed cocyclization of arynes with alkynes: selective synthesis of phenanthrenes and naphthalenes, *J. Am. Chem. Soc.*, **121**, 5827-5828 (1999).
4. Kabalka, G. W.; Li, N.-S.; Pace, R. D. *N*-​*t*-​butoxycarbonyl protection of primary and secondary amines in the hydroboration reaction: synthesis of amino alcohols, *Synthetic Commun.*, **25**, 2135-2143 (1995).
5. Che, Y.; Datar, A.; Balakrishnan, K.; Zang, L. Ultralong Nanobelts Self-Assembled from an Asymmetric Perylene Tetracarboxylic Diimide. *J. Am. Chem. Soc.* **129**, 7234-7235 (2007).
